# Supplementary material for: Pointwise error estimates in localization microscopy
Source: Nat Commun. 2017 May 3;8:15115. doi: 10.1038/ncomms15115 (PMC5418599; doi:10.1038/ncomms15115)
Supplement: Supplementary Information — Supplementary Figures, Supplementary Notes and Supplementary References. [file ncomms15115-s1.pdf]

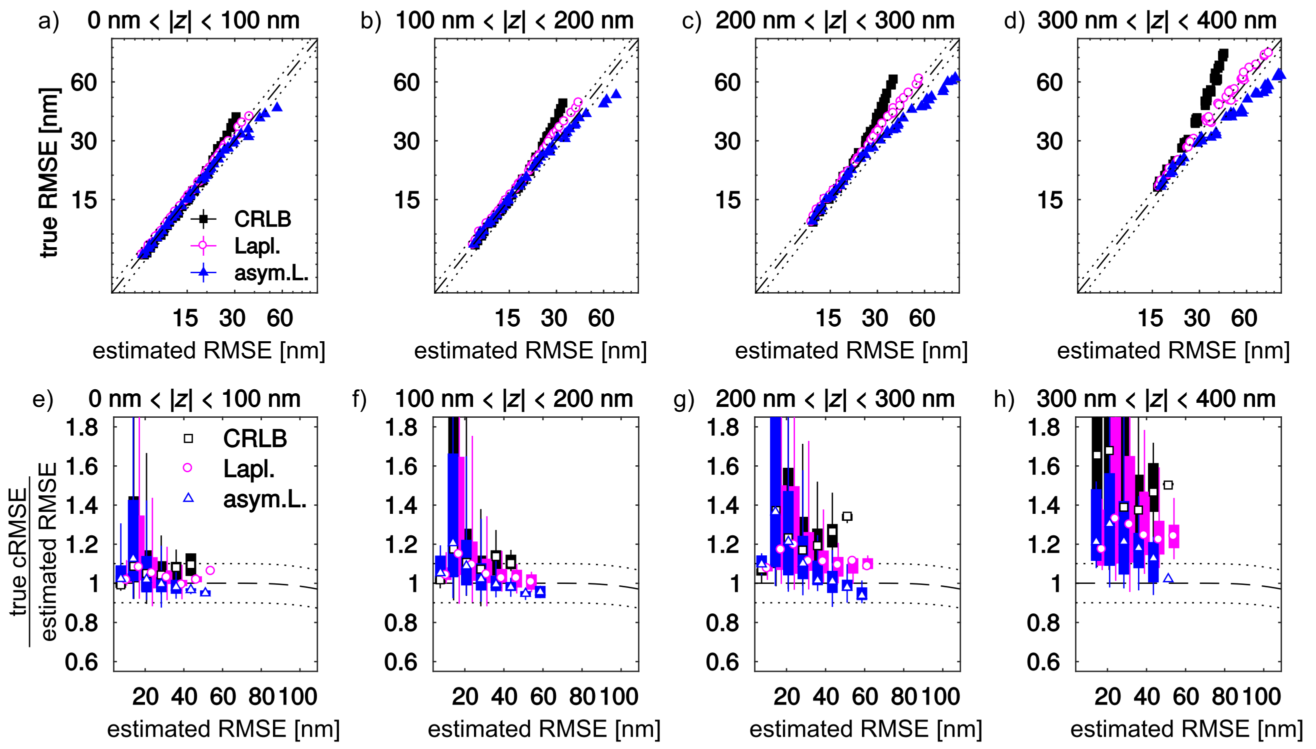

Supplementary Figure 1: Influence of defocus on maximum likelihood (MLE) fits. To illustrate the role of defocus, we subdivide the MLE fits according to their average absolute defocus $|z|$, where z is the z-coordinate averaged over the exposure time (extracted from the simulations). Because the spot width depends on z, the overall errors increases with increasing defocus. More interestingly, the difference between the three estimators also vary strongly with defocus, and all estimators perform quite well for the most well-focused spots. However, the conditional precision estimates still show significant bias at small precisions, since we did not filter on spot width in these graphs (as explored in Supplementary Note 1). Analysis and visualization mimic those in Fig. 2a,b. (a-d): true and estimated root-mean-square error (RMSE). (e-h): Normalized conditional RMSE. In both cases, spots are sorted according to their average z-position (averaged over the exposure time). Exposure times 1 ms,3 ms, and 6 ms.

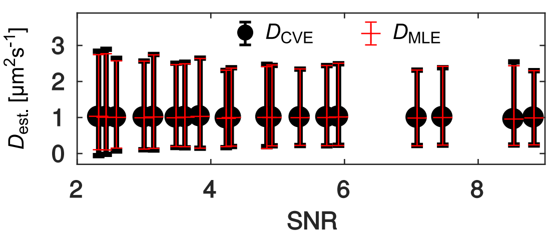

Supplementary Figure 2: Mean value and 1% quantiles of estimated diffusion constants using the covariance-based diffusion constant estimator (CVE) of Ref. [1] and Supplementary Note 9, and the maximum likelihood diffusion constant estimator (MLE) of Supplementary Note 10. The data is the same as in Fig. 5, and the true diffusion constant (still) 1 $\mu m^2 s^{-1}$. Both the estimators use position and precision data, but the MLE explicitly uses the estimated uncertainty of individual positions, while the CVE only uses averages, but the results are almost indistinguishable. However, the fact that the MLE estimator is limited to return positive estimated diffusion constants and handles missing data points (by assigning them infinite or very large variances) may be useful in practice. On the other hand, the CVE is both simpler to implement and significantly faster.

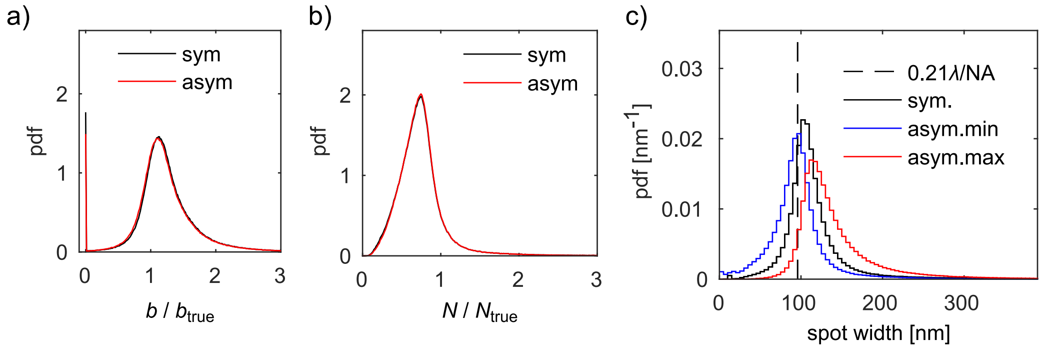

Supplementary Figure 3: MLE fit parameter distributions (empirical probability density functions, pdf) from symmetric and asymmetric fits. a) Normalized background, b) normalized spot amplitude, c) spot width, with the theoretical minimum width $\sigma_0 = \frac{0.21\lambda}{\mathrm{NA}}$ marked by a vertical dashed line. For the asymmetric fits, the distribution of largest and smallest width are shown. Exposure times 1 ms,3 ms, and 6 ms.

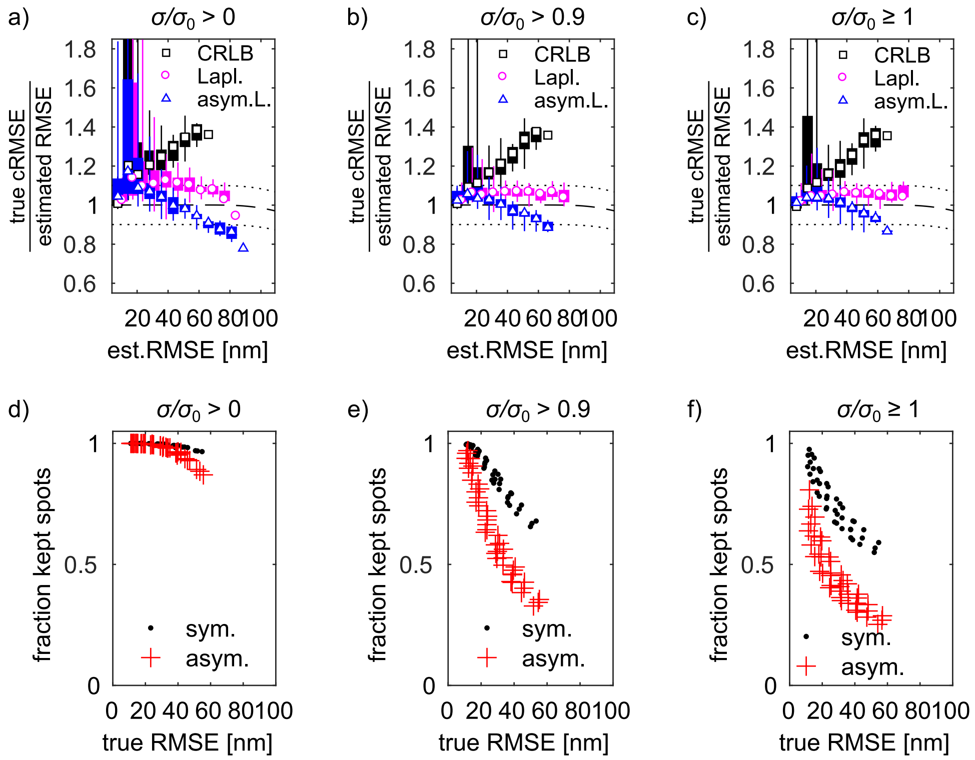

Supplementary Figure 4: Spot width thresholds on maximum likelihood (MLE) fits. (a-c): Normalized conditional root-mean-square errors (RMSE) for various thresholds on the relative spot width, σ/σ_0, where $\sigma = 0.21\lambda/\mathrm{NA}$ is the minimum spot width. Laplace estimates are shown using both a symmetric and asymmetric Gaussian spot model, while the Cramér-Rao lower bound (CRLB) estimates use the symmetric fit. For the asymmetric fits, the threshold is applied to the smaller width parameter. (d-f): Fraction of spots passing the threshold. Data comes from simulated exposure times of 1 ms,3 ms, and 6 ms.

Supplementary Note 1 Sub-populations in MLE fits

Here, we look at some subpopulations of MLE localized spots, with the aim to get further insight into what goes wrong in the MLE precision estimates, and to look at the effect of various selection thresholds (or aposteriori filters).

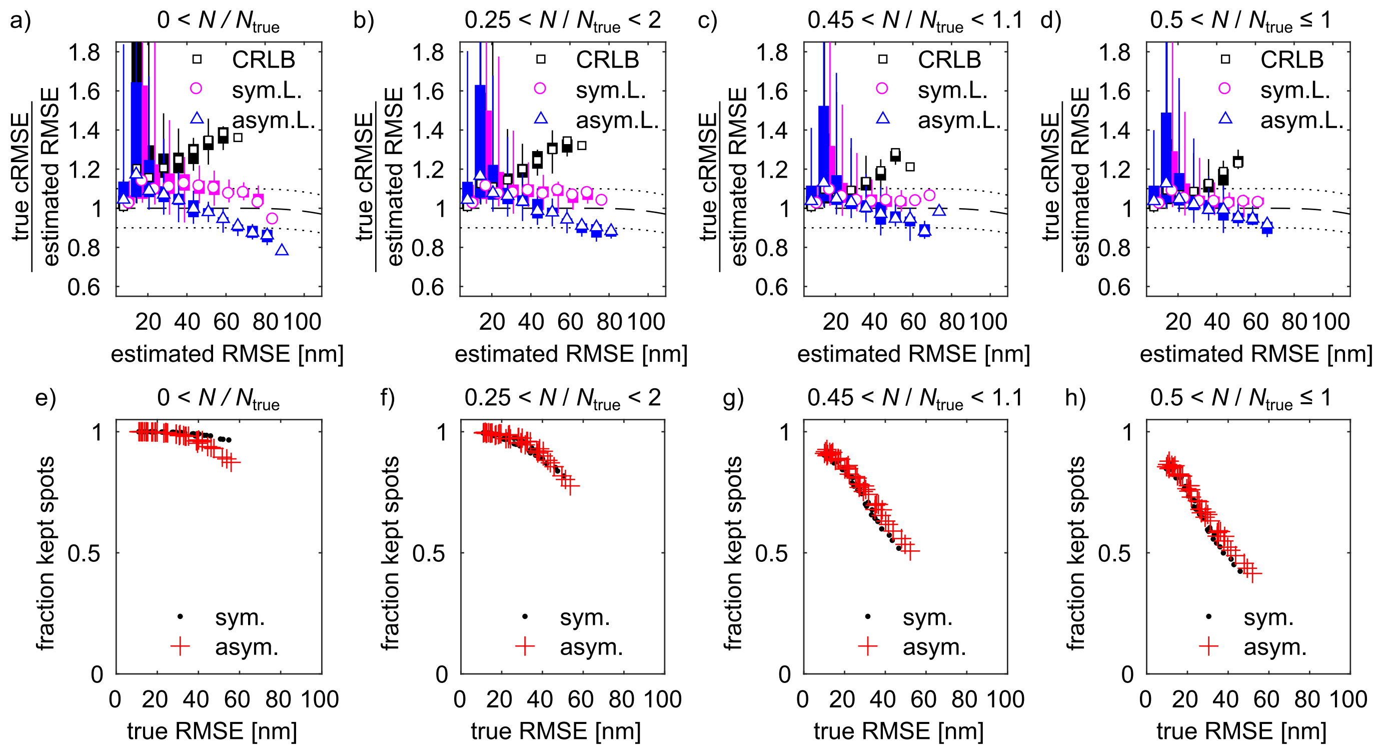

Supplementary Figure 5: Spot amplitude thresholds and maximum likelihood (MLE) fits. (a-d): Normalized conditional RMSE for various thresholds on the relative spot amplitude N/N_{true}. (e-h): Fraction of spots passing the thresholds. Data comes from MLE fits, exposure times 1 ms,3 ms, and 6 ms, and is plotted as in Supplementary Figure 4.

Parameter distributions

The full fit parameter distributions are shown in Supplementary Figure 3. Overall, the distributions are quite broad, and in the case of background and spot amplitude, not centered on the true (simulated) value. As we will see below (and was noted also in Ref. [2]), this bias is intrinsic to the Gaussian spot model. We also note a subpopulation of fits with almost zero background both for the symmetric and asymmetric spot models. For the spot width, a significant portion of the fits end up below the theoretical minimum width $\sigma_0 = \frac{0.21\lambda}{\text{NA}}$, and this portion is larger for the asymmetric spot model.

It is natural to think that the fits that deviate most from the true values might be worse in some sense, perhaps associated with bad precision estimates. This could be translated to a simple parameter filter, where spots with “extreme” fit parameters are discarded. To test this, we apply parameter thresholds on the background, spot amplitude, and spot width, and see how this affects the conditional RMSE estimates and on the fraction of spots that are kept.

For the spot width, we use a lower threshold only, which we set to some (varying) fraction of the minimum width σ_0. As shown in Supplementary Figure 4, this does improve the consistency of the conditional RMSE, and also weakens the tendency to underestimate the precision at low errors. However, this comes at the price of discarding a quite large fraction of the spots, especially when the average RMSE is high.

For the spot amplitude, we use both a low and high threshold, but keep the lower threshold below half of the true value to account for the fact that the amplitude estimator is biased (see Supplementary Figure 8 below). The result, shown in Supplementary Figure 5, resembles that of the spot width: the consistency of the precision estimates is improved, but at the cost of discarding a significant fraction of the spots. Also, the effect on the bias at low errors is weaker than for the spot width threshold.

For the background, we also use both a lower and upper threshold. As shown in Supplementary Figure 6, this works less well. The consistency of the precision estimate does not improve as much as for the width and amplitude thresholds, although the fraction of discarded spots are at least as high. This might be partly because the precision is less sensitive to the background (at least at the background levels we use here), but also because the bias of the

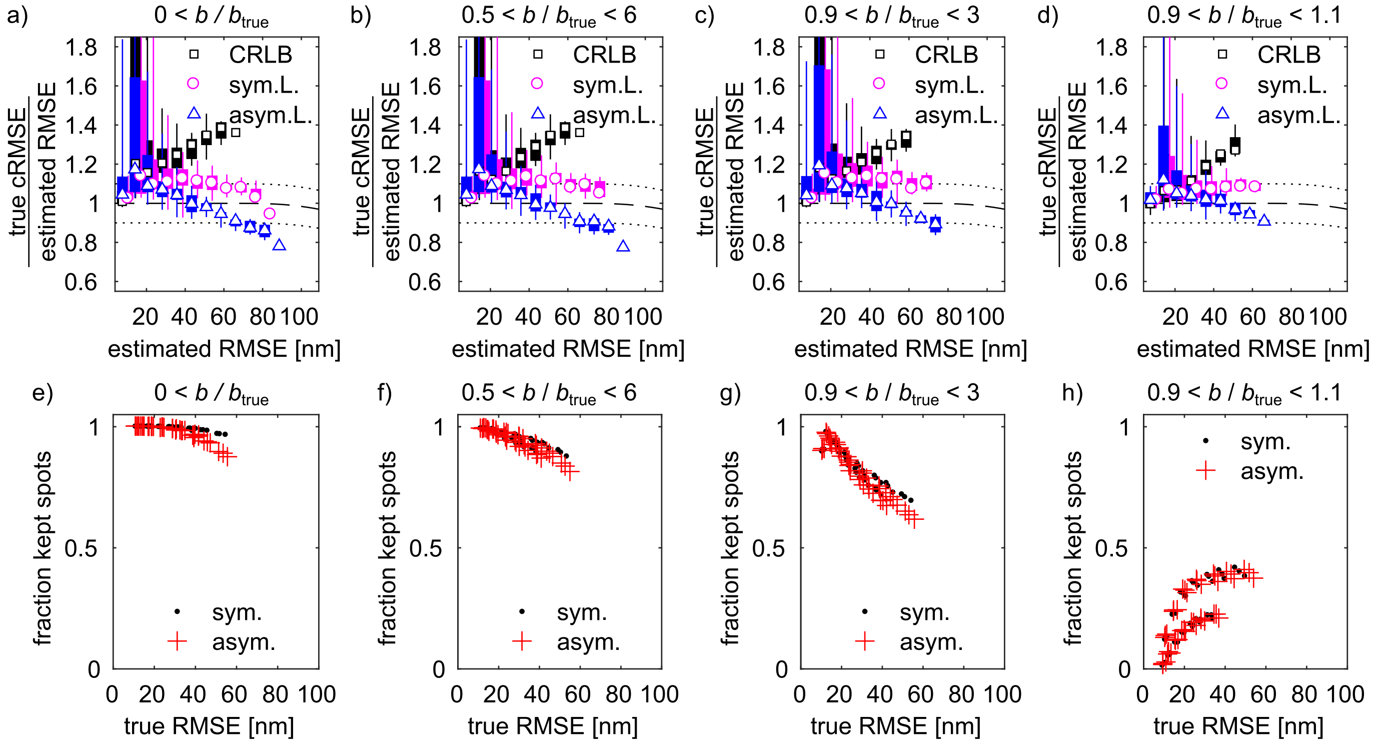

Supplementary Figure 6: Background thresholds and maximum likelihood (MLE) fits. (a-d): Normalized conditional RMSE for various thresholds on the relative background intensity b/b_{true}. (e-h): Fraction of spots passing the threshold. Data comes from MLE fits, exposure times 1 ms,3 ms, and 6 ms, and is plotted as in Supplementary Figure 4.

background estimator depends on the relation between background and spot amplitude (see Supplementary Figure 7 below), which makes it more difficult to define a good general threshold.

Implications

Summing up, the main reason for the bad performance of the CRLB on our test data is that it is less robust towards defocused spots than the Laplace approximation. Second, we see indications that the inconsistency of the conditional RMSE estimates at small errors is mainly due to a sizable subpopulation of spots where the MLE fits converge to a model with unphysically low spot width. Attempts to improve precision estimates by thresholding on fitted spot width and spot amplitude seems to work, but in a large range of conditions, the number of discarded spots is too large to make it an attractive method in practice.

Single particle tracking is especially sensitive to discarded fits, since this breaks up trajectories into shorter pieces and limits the range of dynamics that can be observed. In addition, it can introduce bias in the downstream analysis, since spot quality often correlates with biologically interesting properties such as the diffusion constant.

Part of the problem with parameter thresholds is that in low light conditions, the statistical fluctuations of the fit parameters are larger than the systematic effects, which makes thresholds unnecessarily wasteful. Another difficulty is that the Gaussian spot models are not unbiased estimators of background and spot amplitude (see Supplementary Note 2), so even if those are known exactly (as is the case here, but not in real applications), good thresholds may not be so easy to define.

Supplementary Note 2 High-intensity spot simulations

The distributions of fit parameters from the localizations have two different sources: sampling fluctuations and intrinsic image fluctuations. Sampling fluctuations are random effects of the fact that we are fitting a model to an image with a fairly low number of randomly emitted photons and in the presence of electronic camera noise, and are expected to decrease with increasing number of photons in each image. Intrinsic image fluctuations refer to the fact

that the fluorophore moves randomly during exposure, and hence images of different spots still look different even if we had an infinite number of photons. In live cells, other sources of sample heterogeneity also contribute to intrinsic fluctuations.

The intrinsic fluctuations can be thought of as a concrete realization of a prior distribution (a simple and instructive example is described in Ref. [3]), and empirical Bayes approaches that learn prior parameters from the data along with individual fit parameters have been developed for some single molecule techniques [4, 5].

Here, start instead with an exploratory approach, and analyze simulated data where the statistical fluctuations have been suppressed by increasing the overall intensity of the images. Concretely, we run diffusion simulations with $N = 12\,000$ photons per spot, background intensities ranging from 20-320 photons per pixel, readout noise 0.1, and EM gain 10 (to avoid numerical overflow in the simulated images). To relate this to experimentally relevant parameters, it is useful to compare the relative spot and background intensities. We therefore define the amplitude-background ratio (ABR) as the average photon density inside one standard deviation of a diffraction limited spot compared to the average background intensity,

$$\mathrm{ABR} = \frac{0.3935N/(\pi\sigma_0^2)}{b/a^2}, \tag{1}$$

where $a = 80$ nm is the pixel size, b is the background (photons per pixel), $\sigma_0 \approx 0.21\lambda/\mathrm{NA}$ is the std. of a diffraction limited spot, and

$$P(|\mathbf{x}| < 1|\mathbf{x} \sim N(\mathbf{0}, \mathbf{1})) = \int_0^1 re^{-r^2/2}\mathrm{d}r \approx 0.3935 \tag{2}$$

is the probability mass inside 1 standard deviation in a 2D Gaussian distribution. Our experimentally relevant numbers, $N = 100 - 600$ photons and $b = 1, 3$ photons per pixel, thus have ABR $= 3 - 50$, which is also the range we use for our high-intensity simulations.

Results with symmetric Gaussian spot model

Supplementary Figure 7 and Supplementary Figure 8 show distributions of background and spot amplitude from high-intensity simulations at various exposure times. We see large bias in both parameters, downwards and mainly ABR-independent for the spot amplitude, and increasingly upwards with increasing ABR values for the background intensity. This behavior can be largely understood in terms of two effects. First, our simulations contain a mixture of spots in and out of focus. Defocused spots increasingly spread photons outside the 9-by-9 region of interest we use for fitting, leading to some downward bias in spot amplitude. More importantly, real PSFs have "shoulders" which are not well described by Gaussians spot models [2]. As a consequence, our localization procedure misinterprets some of the spot intensity as increased background. This contributes to a downward bias in spot amplitude, and also explains why the relative background bias is largest when for high relative spot intensity. An amplitude-background scatter plot (Supplementary Figure 9) also reveals ABR-dependent correlations between amplitude and background, as well as a small population of spots with essentially zero background.

Supplementary Figure 10 show spot amplitudes, normalized by the std. σ_0 (see Eq. 1) of a diffraction limited Gaussian spot. The spot widths scatter in a limited interval above σ_0, showing that the fits below this limit in the low-light simulations (Fig. 2d) are indeed fit errors. The shape of the distributions show no strong dependence on ABR, but instead group depending exposure time t_E. This is a motion blur effect, which is more appropriately understood in terms of the diffusion length during exposure, which scales as $\sqrt{2Dt_\mathrm{E}}$. Thus, data with multiple diffusive states, which is a biologically relevant case for single particle tracking experiments [6], will contain a mixture of spot width distributions. Moreover, the widening of the spot images is both due to in-plane motion and the z-dependence of the PSF. The compound net effect therefore also depends on the imaging wavelength and the sample geometry, as illustrated from the quite different spot width distribution when the wavelength is decreased from 369 nm to 514 nm. For example, $t_\mathrm{E} = 22$ ms at 639 nm is quite similar to $t_\mathrm{E} = 10$ ms at 514 nm.

Results with asymmetric Gaussian spot model

We also analyzed the high intensity 639 nm simulations with an asymmetric Gaussian spot model, Eq. (10). The background and spot amplitude distributions are essentially identical to those from the symmetric model, but each fit generates two spot widths, whose distributions are showed in Supplementary Figure 11.

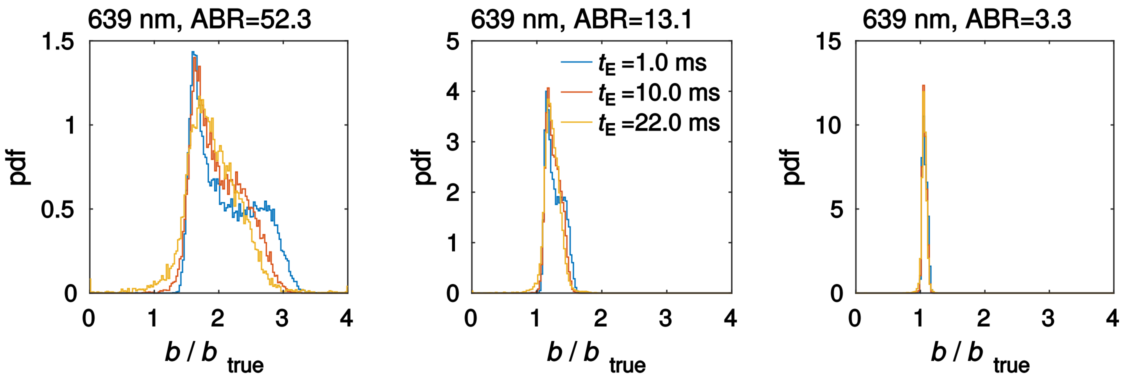

Supplementary Figure 7: Probability density function (pdf) of rescaled background intensity $b/b_{\rm true}$ for various exposure times and amplitude/background ratios, at λ = 639 nm.

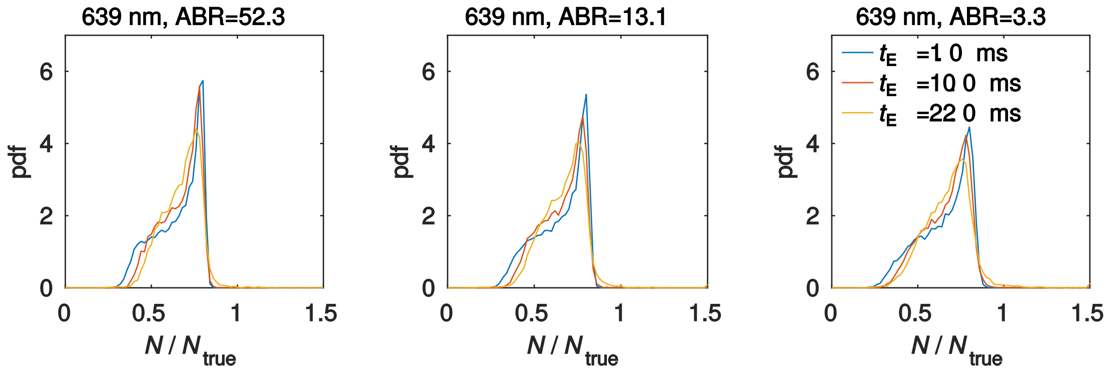

Supplementary Figure 8: Probability density function (pdf) of rescaled spot amplitude $N/N_{\rm true}$ for various exposure times and amplitude/background ratios, at λ = 639 nm.

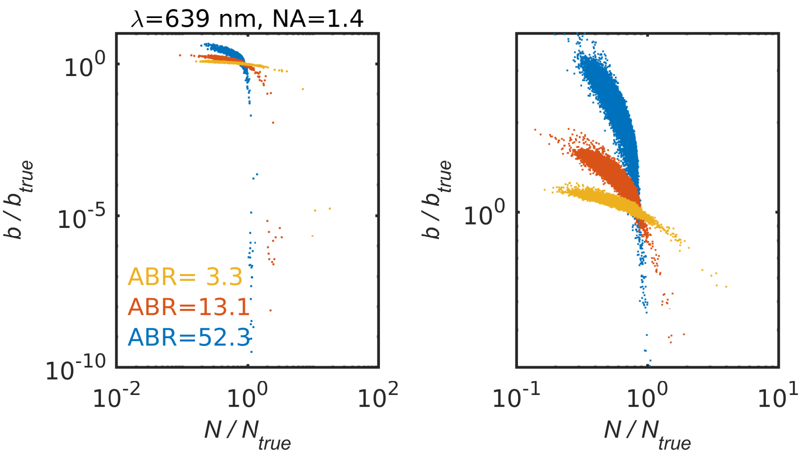

Supplementary Figure 9: Scatter plots of rescaled spot amplitude and background, sorted on amplitude/background ratio. Both panels show the same data in different scales.

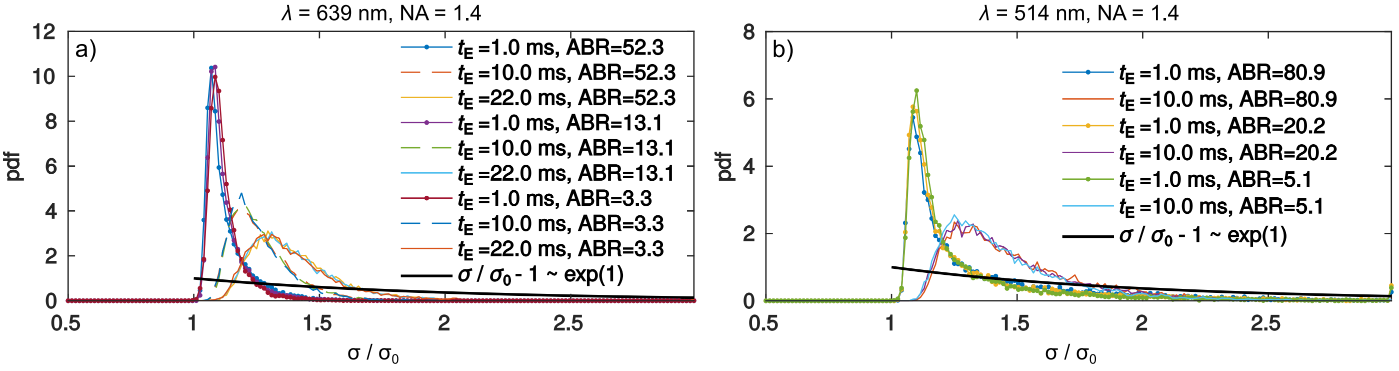

Supplementary Figure 10: Probability density function (pdf) of rescaled spot width σ/σ_0, for various exposure times t_E and amplitude/background ratios (ABR), with wavelength (a) $\lambda = 639$ nm and (b) $\lambda = 514$ nm. Fit parameter refers to a symmetric Gaussian spot model. Also shown is the exponential prior on the excess spot width $\sigma/\sigma_0 - 1$.

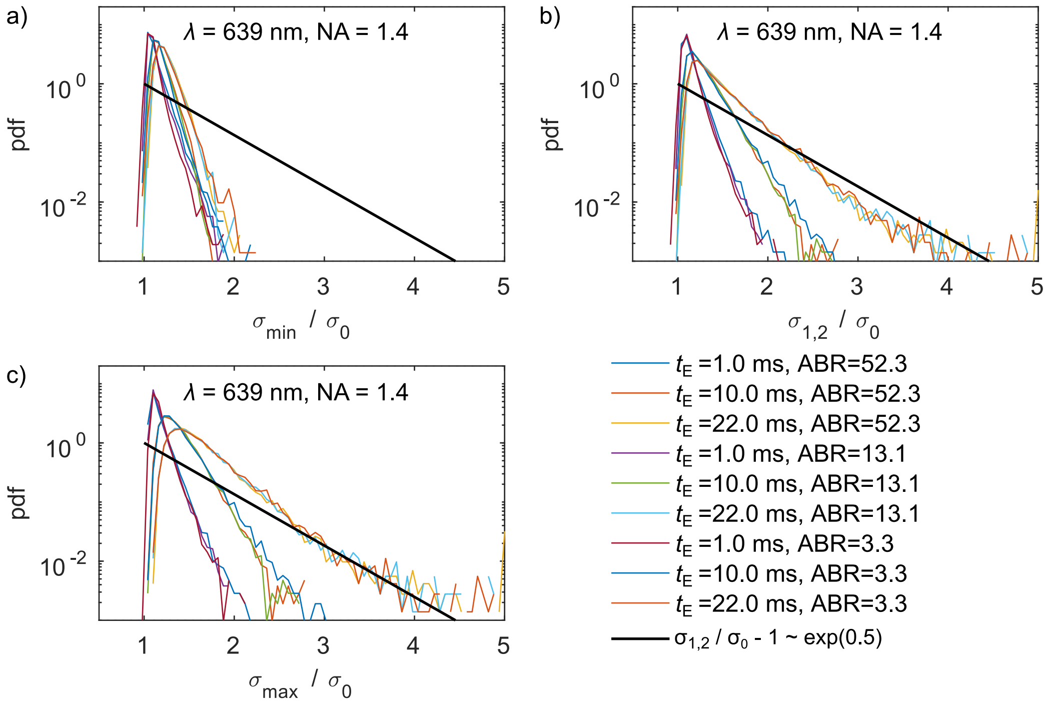

Supplementary Figure 11: Probability density function (pdf) of rescaled spot widths $\sigma_{\ldots}/\sigma_0$ from an asymmetric Gaussian spot model, for various exposure times and amplitude/background ratios. a) Distribution of smallest width parameter σ_min in each fit. b) Aggregated distribution of both width parameters from each fit. c) Distribution of largest width parameter σ_max in each fit. Also shown is the exponential prior on the excess spot widths $\sigma_{1,2}/\sigma_0 - 1$.

Supplementary Note 3 Localization priors

From the high intensity simulations above, it is clear that the quantitative relation between fit parameters and true parameters depends in a complex way on a range of experimental parameters, not all of which are easy to measure of control accurately in real experiments. We therefore do not attempt to construct a quantitative models for the intrinsic image fluctuations, but instead try a “weakly informative” approach, where we aim merely to exclude unreasonable parameter values (such as $\sigma < \sigma_0$) without exerting strong influence in regions with reasonable values, and also to regularize the numerical problem.

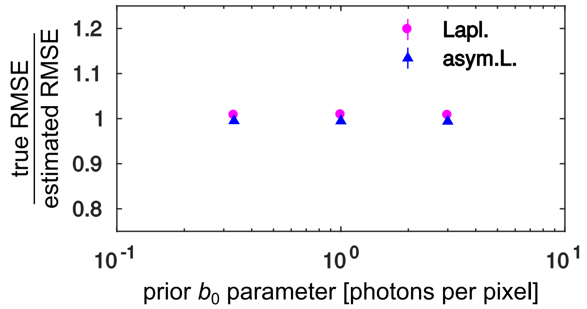

Supplementary Figure 12: Normalized (true / estimate) root-mean-square error (RMSE) for maximum aposteriori estimates (MAP) for spots with 270 photons, 3 ms exposure time, and background 1 photon per pixel, analyzed with different mean value parameter b_0 for the background prior. Results for both symmetric and asymmetric spot models are shown, each run with our standard choice of spot width prior.

Weak regularization of background intensity

For regularization, we notice that the background intensity in some cases gets very low, b on the order of e^{-10} or lower, that partial derivatives of the likelihood w.r.t. $\ln b$ are (near) zero. This can make the Laplace precision estimator numerically unstable, since it depends on inverting the Hessian matrix of partial second derivatives. Such situations can be regularized using a weak prior.

We choose a normal prior distribution of $\ln b$ (log-normal on b), with a wide standard deviation parameter of $\ln 30$. The prior mean value parameter needs to be estimated from data, but for such a weak prior, an order-of-magnitude estimate is enough, and we should also not have to worry about systematic bias in the background fit parameter (Supplementary Figure 7), or about moderate background variations between different samples or over time for example due to bleaching. Supplementary Figure 12 shows the normalized RMSE for a single imaging condition, analyzed with the background prior mean value parameter differing a factor 3 up and down compared to the true background, demonstrating the insensitivity.

Prior on spot width, symmetric spot model

For the spot prior, we enforce $\sigma \geq \sigma_0$. We would also like to include some upper limit for the spot width, since the high intensity spot width distributions are clearly bounded, and since very wide spots are difficult to distinguish from only background on a finite region of interest.

To enforce a lower bound $\sigma \geq \sigma_0$ for localizations, we re-parameterize the spot model, and write

$$\sigma = \sigma_0(1 + \Delta\sigma) = \sigma_0(1 + e^{\psi}). \tag{3}$$

According to the transformation rules for probability distributions, a prior distribution p_Δ on $\Delta\sigma$ translates to a prior

$$p_\psi(\psi) = p_\Delta(e^\psi)\left|\frac{d\Delta\sigma}{d\psi}\right| = p_\Delta(e^\psi)e^\psi \tag{4}$$

on ψ. If p_Δ is finite as $\Delta\sigma \to 0$ (or at least diverges slower than linearly in $\Delta\sigma$), it will thus stop ψ from taking arbitrarily large negative values, which is good numerically. Similarly, we see that the factor e^ψ can encourage a large parameter value grow without bound, which might cause problems for low-light spots or false positives, where the data might not enforce an upper bound. It therefore makes numerical sense to make the prior counteract this as well.

We find reasonable performance by choosing an exponential prior on $\Delta\sigma$, which means

$$p_{\text{sym}}(\psi) = \frac{1}{d_0}\exp\left(\psi - \frac{e^\psi}{d_0}\right), \tag{5}$$

where d_0 should be of order unity, so that the prior is close to flat in the most occupied parameter region in Supplementary Figure 10. Trying a few different d_0 values (Supplementary Figure 13) we see some dependence on d_0, and choose to continue with $d_0 = 1$.

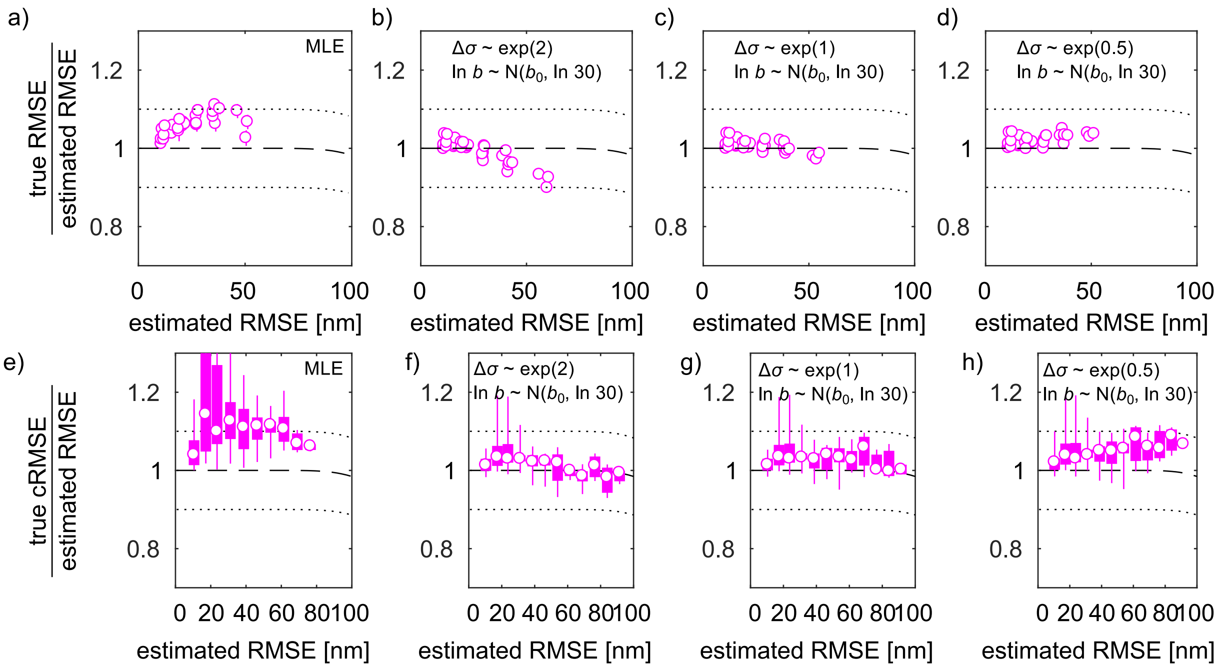

Supplementary Figure 13: Different exponential priors on $\Delta\sigma$, with 639 nm wavelength, and 1 ms, 3ms, and 6 ms exposure time. All data is for Laplace precision estimate using symmetric a spot model and a weak log-normal background intensity prior centered on the true background. (a-d) Estimated root-mean-square error (RMSE), normalized by the true RMSE to make the differences more visible. (e-h) Conditional RMSE normalized by estimated RMSE. Dashed and dotted lines show 0, ±10% bias, respectively.

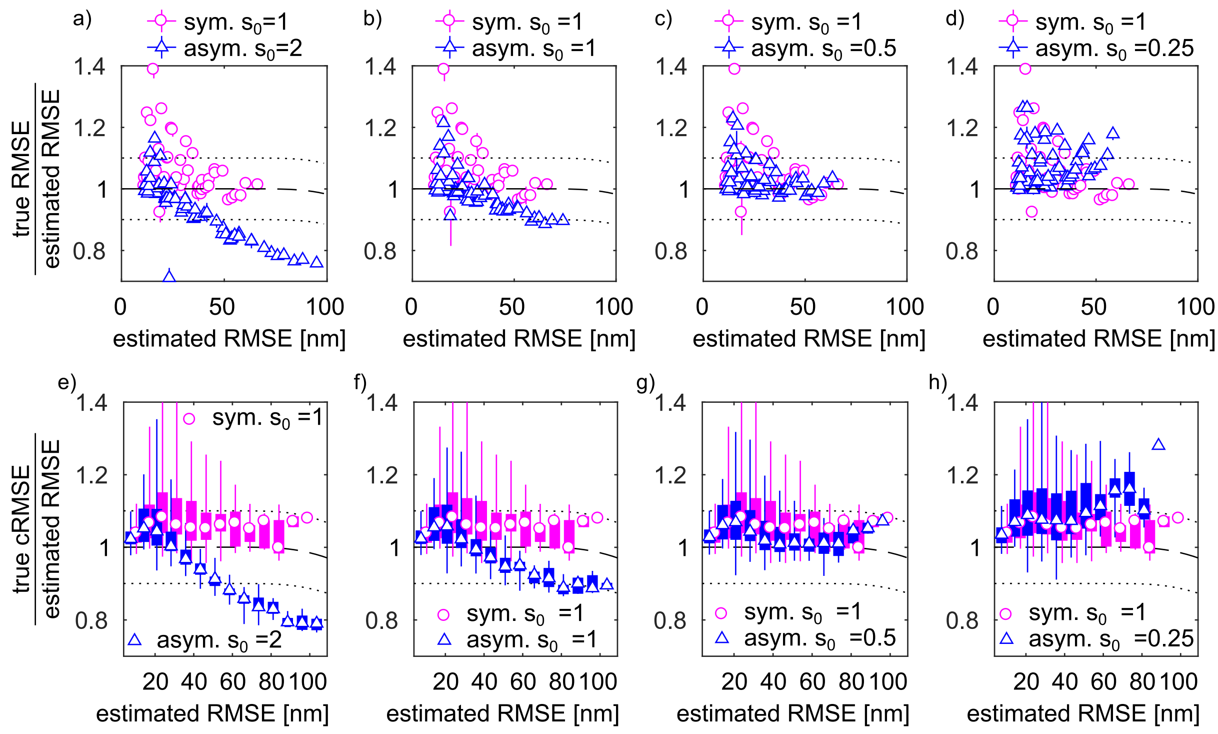

Supplementary Figure 14: Different exponential priors on $\Delta\sigma_i \sim \exp(s_0)$ for the asymmetric spot model, with 639 nm wavelength, and 1-22 ms exposure time. All data is for Laplace precision estimate and a weak log-normal background intensity prior centered on the true background, with the results for the symmetric spot model with the standard $\Delta\sigma \sim \exp(1)$ prior (and weak log-normal background) shown for comparison. Dashed and dotted lines show 0, ±10% bias, respectively.

Prior on spot width, asymmetric spot model

To generalize the above results to an asymmetric spot model we make the corresponding reparameterization

$$\sigma_{1,2} = \sigma_0(1 + \Delta\sigma_{1,2}) = \sigma_0(1 + e^{\psi_{1,2}}), \tag{6}$$

and use independent exponential priors on $\Delta\sigma_{1,2}$,

$$p_{\text{asym}}(\psi_1, \psi_2) = \frac{1}{d_0^2} \exp\left(\psi_1 + \psi_2 - \frac{e^{\psi_1} + e^{\psi_2}}{d_0}\right). \tag{7}$$

Again trying a few different d_0 values on a wider range of exposure times (Supplementary Figure 14), we see a stronger dependence on d_0 than for the symmetric spot model. The best performance, better than for the symmetric MAP fit, clearly is the choice $d_0 = 1/2$ in this case. However, even though the performance is improved, the conditions with significant blur and high spot intensity give quite large relative errors, perhaps because these conditions produce most spots with clearly non-Gaussian intensity profiles.

Curiously, $d_0 = 1/2$ is also when the prior for the symmetric special case $\psi_1 = \psi_2$ agrees best with that of the symmetric prior. Another possible explanation is that as seen in Supplementary Figure 11, the exp(0.5) distribution describes the tail of the larger spot width parameter quite well. Since large values of the largest spot width are likely not well constrained by the data, it makes sense that the performance of the model is best when this part is well described by the prior. If this is the case, the asymmetric spot model with this prior may work less well in conditions where the spot width tails behave differently. Lesser robustness of the asymmetric prior can also be seen by comparing the d_0-dependence of the symmetric and asymmetric models, and note that a factor 2 difference around the optimal value makes a larger impact in the asymmetric (Supplementary Figure 14) than the symmetric (Supplementary Figure 13) case.

EM gain calibration

The likelihood function we use for localization depends on the camera noise parameters, and miscalibration of those parameters may influence the result. In particular, one suspects that the EM gain parameter is important, since it is the average conversion factor between image intensity and number of photons, and estimation errors depend strongly on the number of photons. Supplementary Figure 15 shows the effect of gain calibration errors. The true RMSE appears insensitive, but the estimated precision does depend on the gain used in the localization.

To understand this dependence, we note that the estimated errors scales with the number of photons as $RMSE \propto 1/\sqrt{N_{photons}}$, and that the apparent number of photons is inversely proportional to the gain, $N_{photons} \propto 1/g$. Combining the two, we conclude that the estimate RMSE should scale as $\sqrt{g/g_0}$, where g_0 is the true gain, and g is the gain used for localization. As seen in Supplementary Figure 15a, this relation seems to describe the estimated RMSE pretty well.

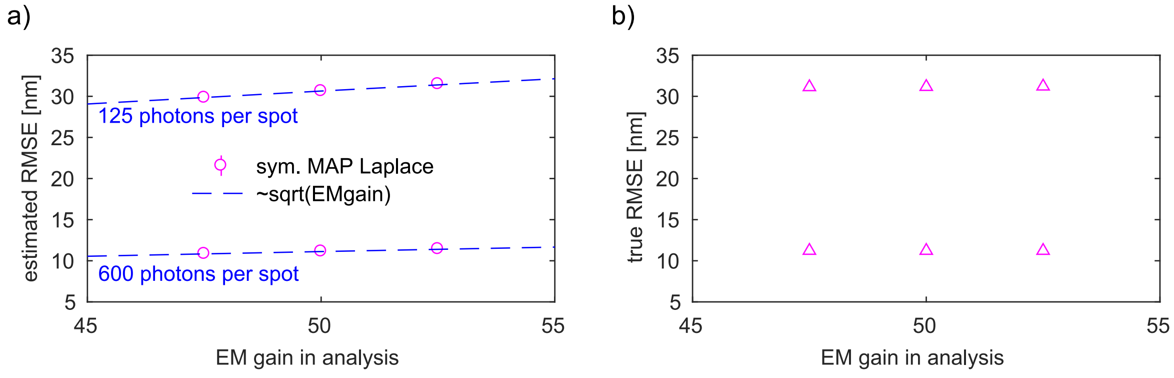

Supplementary Figure 15: Effect of EM gain calibration error. a) Estimated root-mean-square error (RMSE). Dashed lines illustrate the scaling proportional to $\sqrt{gain}$. b) true RMSE. Precision was estimated using the symmetric MAP Laplace estimator. All spots were simulated with EM gain 50, but analyzed with 0, ±5% calibration error in the EM gain. Misc. spot parameters: 125 or 600 photons per spot, background 1 photon per pixel, 3 ms exposure.

Supplementary Note 4 Influence of priors on localization errors and spot convergence

Aside from influencing the precision estimates, the priors may also influence the actual localization errors. Such a comparison is shown in Supplementary Figure 16, for both MLE and MAP localization, using both symmetric and asymmetric Gaussian spot models, with symmetric MLE localization used as the reference case on the x axis. The localization errors of the different methods seems to order as asymmetric MLE (worst) > symmetric MLE > symmetric MAP > asymmetric MAP. However, the differences are no larger than 10%, and all methods seem to perform equally well for the brightest spots (smallest errors).

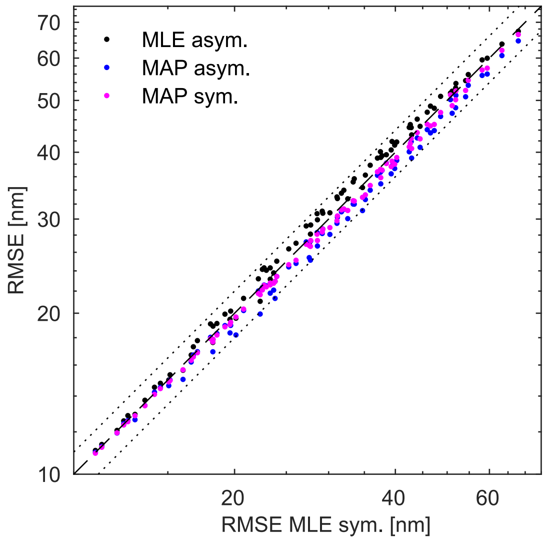

Supplementary Figure 16: Comparing localization errors of the different methods. Root-mean-square error (RMSE) for symmetric vs. asymmetric Gaussian spot models and maximum likelihood (MLE) vs. maximum aposteriori (MAP) estimates, with symmetric MLE localizations as reference on the x axis. Simulated images are 639 nm wavelength, exposure times 1-22 ms. Dashed and dotted lines show $y = x$, and ±10% change, respectively.

Similarly, the priors affect the numerics of the localization problem and overall convergence rates. As seen in Supplementary Figure 17, the MAP localization leads to an increased fraction of kept spots. This is an expected effect of trying to repress fits that lead to badly conditioned Hessian matrices.

However, the performance difference on real data might be different, since the spots included in our localization tests are selected based on known true positions. Thus, the complications of spot detection, which would most likely lead to a quite different population of candidate spots for localization, are not accounted for here (however, see Supplementary Note 6).

Supplementary Note 5 PSF model mismatch

It is tempting to speculate that some of the difficulties with the MLE estimates is because the Gaussian spot model used for localization is not an accurate enough description of the real PSFs. Could one do better by adopting more realistic spot models, perhaps directly parameterized from experimental PSFs [7]?

To test this, we modified our simulations to use a Gaussian PSF instead, thus achieving perfect agreement between fit model and the generative PSF, and tested our precision estimators on this data. To retain some defocus effects, we adopted the model of Deschout et al [8], in which the standard deviation of the Gaussian PSF varies with the distance z from the focal plane as

$$\sigma(z) = \sigma_0\sqrt{1+\frac{z^2}{z_0^2}}, \quad z_0 = \frac{4\pi n}{\lambda}\sigma_0^2, \tag{8}$$

where σ_0 is the spot width at the focal plane and n is the refraction index. We used $\lambda = 639$ nm and NA $= 1.4$, and $n = 1.33$, and Eq. (1) to compute σ_0, which gave $\sigma_0 \approx 96$ nm and $z_0 = 489$ nm. For the simulations, we used the same backgrounds, spot amplitudes, and exposure times as with the more realistic PSFs used for Figs. 2 and 3, and also used the same analysis procedures.

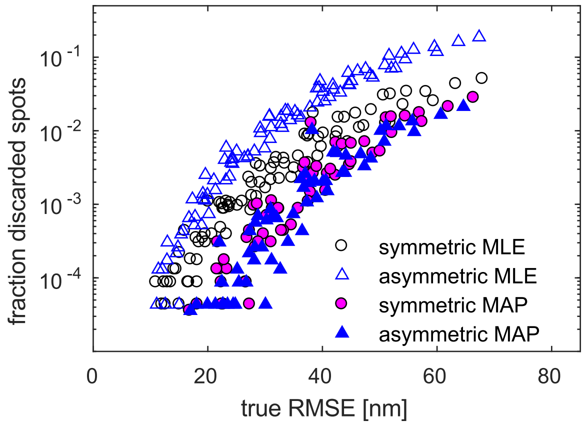

Supplementary Figure 17: Comparing the fraction of discarded spots as a function true root-mean-square error (RMSE) symmetric vs. asymmetric Gaussian spot models and maximum likelihood (MLE) vs. maximum aposteriori (MAP) estimates. Simulated images are 639 nm wavelength, exposure times 1-22 ms.

The results are shown in Supplementary Figure 18. Qualitatively, there is a striking similarity to the results using the more realistic PSFs (Figs. 2 and 3), and we recognize all major bias trends described in the main text. In more detail, the Gaussian PSF simulations yield some quantitative improvements: the largest errors are somewhat smaller, as is the the median bias of the conditional RMSEs, and the interval lengths in the conditional RMSE box plots are also somewhat more narrow. These improvements are quite modest however. They are also a little uncertain: Since the overall errors are also smaller for Gaussian PSF data, the comparison might not be completely fair.

Theoretically, it is perhaps not so surprising that localizing with a spot model that perfectly matches the PSF gives no dramatic improvement. Even for our fairly short exposure times, the fluorophores do move during exposure, and thus the actual spot profiles are not perfect Gaussians even if the PSF is.

Supplementary Note 6 Consequences of spot detection

So far, we selected ROIs based on the known true positions of the spots, extracted from the simulations. This is not possible for real data, and one might ask if the performance we see is representative of real applications. Two effects are of particular concern. First, using the true positions to select ROIs and initial guess for the localizations might make the optimization problem artificially well-behaved by reducing the risk of getting stuck in local minima, and thereby lead to overly optimistic results. On the other hand, the pool of candidate spots resulting from using the true positions may include spots that would not be detected by a spot detection algorithm, for example because they are far out of focus. Since hard-to-detect spots are likely to be difficult to localize as well, this would tend to make the simulated data artificially ill-behaved.

To estimate how these effects may play out in real data, we ran numerical experiments using a spot detection algorithm to pre-filter out hard-to-detect spots. Specifically, we use a feature detection transform that targets local radial symmetry [9], and also provides spot locations that we use as initial conditions for the localizations. We did not optimize the transform parameters for every imaging condition, but use conservative settings that seem to work well for the middle of our spot intensity range. As a result, 25-50% of the most low intensity spots was not detected, and false positive rate was generally low (multiple spots detected in 0-2% of the images). For simplicity, we also discarded spot detections more than 3 pixels away from the true positions as false positives. The remaining spot detections where used as initial conditions for the MLE and MAP localization procedures described above.

Supplementary Figure 19 compares estimated and true RMSE as in the main text, for low blur conditions (exposure time up to 6 ms), with and without the spot detection pre-filter. Several differences stand out. Because of the pre-filter's tendency to discard ill-behaved spots, the overall errors are lower after pre-filtering. The asymmetric MLE fits also perform better on the remaining spots with high localization error, as does the MAP CRLB estimator. However, the bias in precision at low estimated precision remains in the MLE fits, and thus the improved performance of our MAP estimators is clear also in the more well-behaved pre-filtered data set. It also appears that the initial positions provided by the radial symmetry transform work well enough for the optimization, so that the results using true positions for initializations are not over-optimistic in this respect.

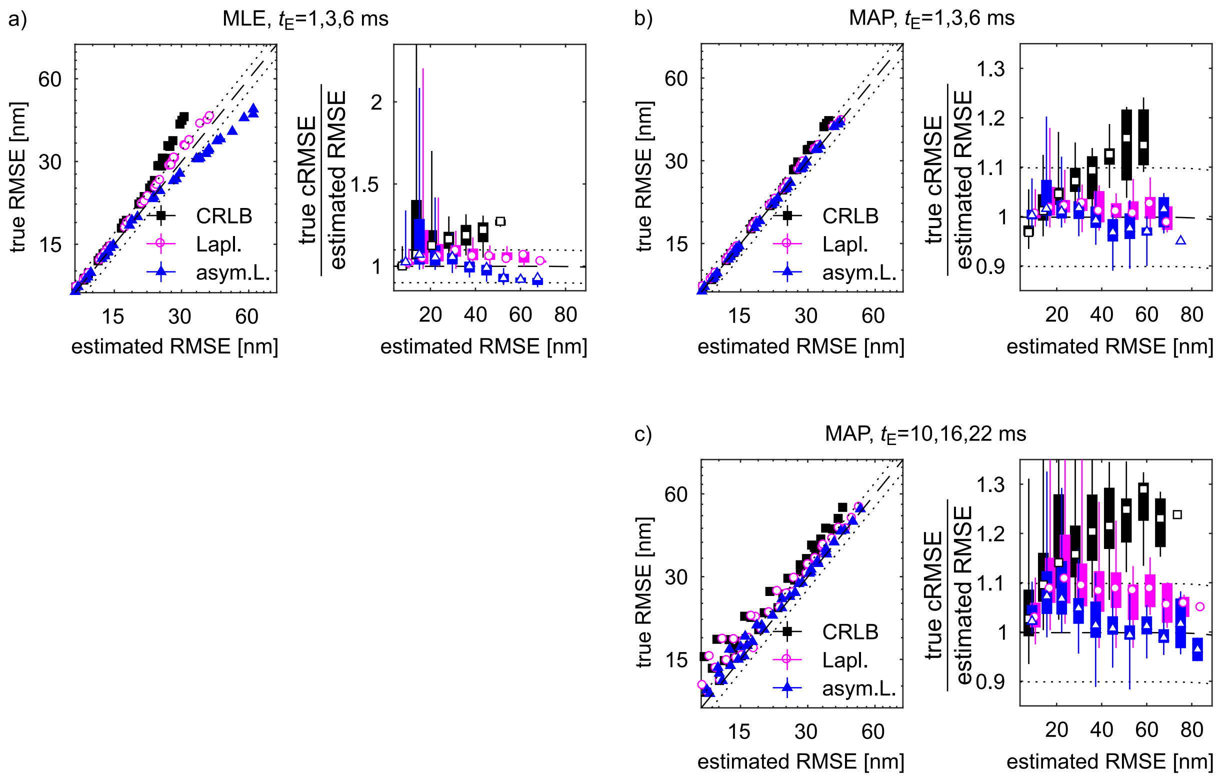

Supplementary Figure 18: True and estimated precision (root-mean-square error, RMSE, and conditional RMSE, cRMSE) using symmetric/asymmetric spot models (asym.), maximum likelihood (MLE)/maximum aposteriori (MAP) localization, and Cramér-Rao lower bound (CRLB)/Laplace (Lapl., L.) precision estimates, on simulated data with a z-dependent Gaussian point-spread function. Dashed and dotted lines show 0, ±10% bias, respectively.

Supplementary Note 7 Spot models for localization

We use two common spot models for localization, based on a constant background intensity plus a Gaussian intensity profile for the spot. We parameterize the symmetric spot in the usual manner,

$$E(x,y) = \frac{b}{a^2} + \frac{N}{2\pi\sigma^2}\exp\Big(-\frac{(x-\mu_x)^2+(y-\mu_y)^2}{2\sigma^2}\Big), \tag{9}$$

with pixel size a (known), background b (expected number of photons per pixel), spot width σ, amplitude N (expected number of photons per spot), and spot position (μ_x, μ_y). For the asymmetric spot model, we use a representation in terms of two principal widths and a rotation angle,

$$E(x,y) = \frac{b}{a^2} + \frac{N}{2\pi\sigma_1\sigma_2}\exp\Big(-\frac{1}{2}(\mathbf{x}-\boldsymbol{\mu})^\dagger\mathbf{R}^\dagger(v)\begin{bmatrix}\sigma_1 & 0\\ 0 & \sigma_2\end{bmatrix}\mathbf{R}(v)(\mathbf{x}-\boldsymbol{\mu})^\dagger\Big), \tag{10}$$

where

$$\mathbf{R}(v) = \begin{bmatrix}\cos v & \sin v\\ -\sin v & \cos v\end{bmatrix} \tag{11}$$

is a rotation matrix. For maximum-likelihood localization (MLE), we use the log of the parameters $b, \sigma, \sigma_{1,2}$ as fit parameters, both as a convenient way to ensure that they stay positive, and because we suspect that their logarithms have more Gaussian-like likelihoods, which would improve the quality of the Laplace approximation [10].

Supplementary Note 8 Spot selection criteria

The choice of what spots to include and exclude in the analysis can impact the results. We select based on 1) an upper threshold on estimated precision, 2) an upper threshold on the distance ΔR between localized position and the center of the ROI, and 3) convergence to parameters where the Laplace precision estimate can be computed, which requires

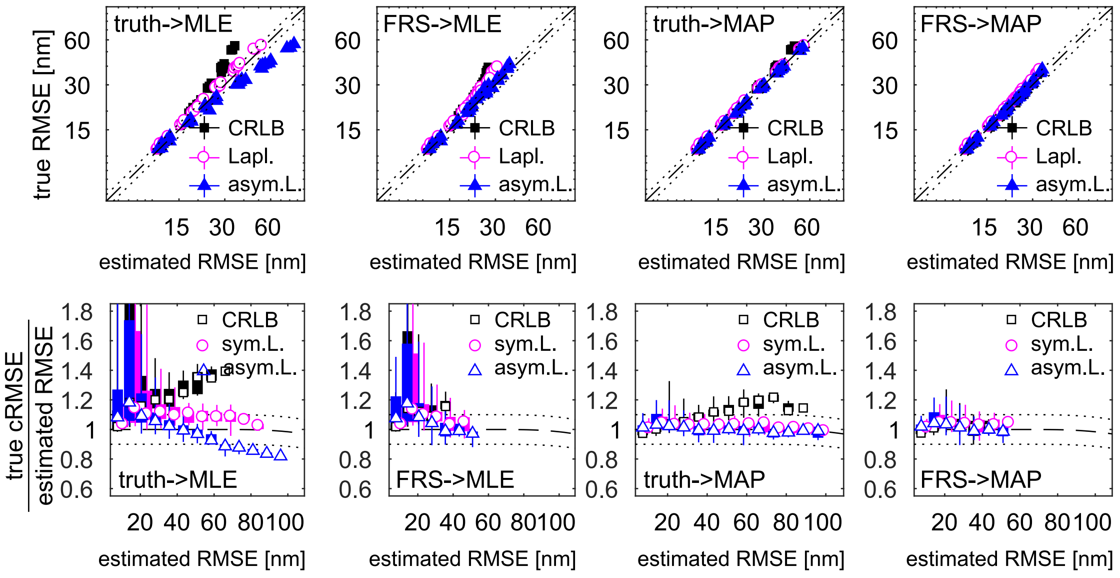

Supplementary Figure 19: True and estimated root-mean-square errors (RMSE) and conditional RMSE (cRMSE) using region of interests and initial spot positions based on true positions (truth) or as detected and localized using with a fast radial symmetry transform (FRS) for spot detection. The localization and precision estimates combines symmetric/asymmetric spot models (asym.), maximum likelihood (MLE)/maximum aposteriori (MAP) localization, and Cramér-Rao lower bound (CRLB)/Laplace (Lapl., L.) precision estimates, using simulated data as described in the main text, with exposure time up to 6 ms. Dashed and dotted lines show 0, ±10% bias, respectively.

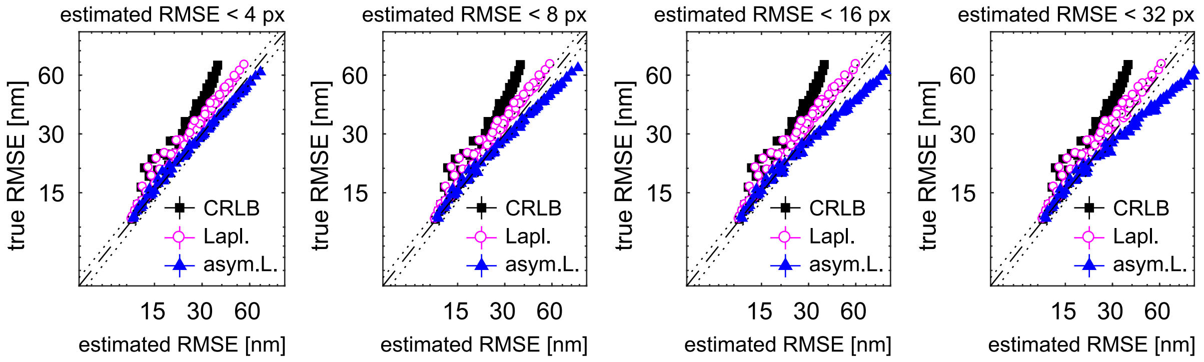

Supplementary Figure 20: True and estimated root-mean-square errors (RMSE) for maximum likelihood (MLE) fits, and various values of the upper threshold on estimated RMSE. The displacement threshold is $\Delta R \leq 4$ pixels = 320 nm, and exposure times range from 1 ms to 22 ms. Dashed and dotted lines show 0, ±10% bias, respectively.

the log likelihood to have an invertible Hessian matrix. (We also have an upper threshold of spot width, which in the case of asymmetric spots are only applied to the smallest principal width, but this affects only a handful of spots).

Supplementary Figure 20 shows a comparison between the true and estimated RMSE for MLE fits for various values of the upper threshold on the estimated RMSE. The data is the same data as Fig. 2, but including all exposure times and both symmetric and asymmetric fits. The Laplace estimators are more sensitive to this threshold than the CRLB. For practical purposes, the lowest 4 pixel threshold is probably reasonable, but since our purpose here is to investigate the consistency, we need converged averages and settle for a 16 pixel threshold. The dependence on the RMSE threshold is much weaker for the MAP fits however, as seen in Supplementary Figure 21.

Next, we consider the effect of the threshold on the displacement ΔR. This cannot be set arbitrarily high, since it makes no sense to retain localizations outside the ROI. However, the effect can be simulated by a simple numerical model which utilizes the fact that our ROI are centered on the true positions: For a given precision ε^2, we simply generate Gaussian localization errors $\Delta\mu_{x,y} \sim N(0, \varepsilon^2)$, and compute the variance of those points that fall inside the

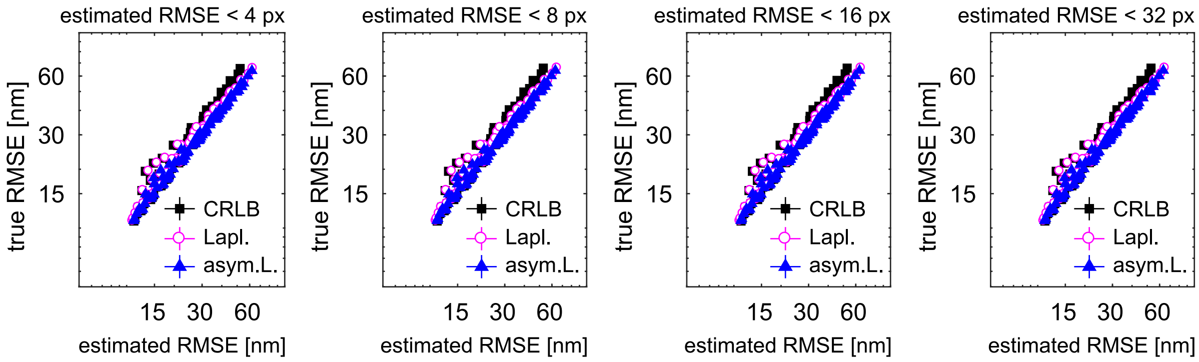

Supplementary Figure 21: Same as Supplementary Figure 20, but for maximum aposteriori (MAP) fits instead.

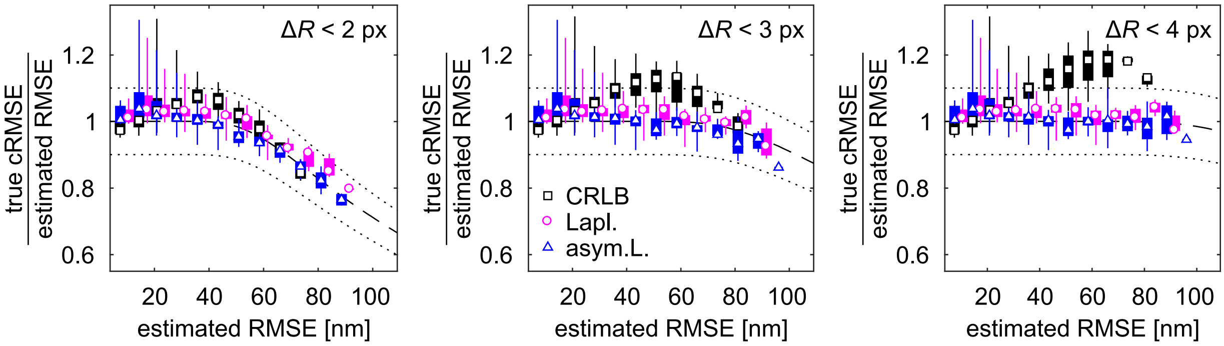

Supplementary Figure 22: Normalized conditional root-mean-square error (cRMSE) for maximum aposteriori (MAP) localizations, using various thresholds on the displacement ΔR. Exposure times 1ms, 3ms, 6ms. Dashed and dotted lines show 0, ±10% bias, respectively.

threshold $\Delta R = \sqrt{\Delta\mu_x^2 + \Delta\mu_y^2} \leq \Delta R_{\max}$. Supplementary Figure 22 compares this model with conditional precision estimated from MAP estimates for short exposure times. The agreement seems quite good, and we also see that $\Delta R_{\max} = 4$ pixel is large enough to effectively remove the artifacts from this threshold.

Supplementary Note 9 Step-length covariance relations with time-varying localization errors

Here, we give a detailed derivation of the various estimators and models mentioned in the methods section, starting from Eq. (8), the assumption that localizing a moving object amounts to detecting the time-averaged position with some (independent) localization error, which we will assume to be Gaussian. Parts of these derivations have been given elsewhere [11, 12], but are restated here in a different form that facilitates a generalization to multi-state models.

Diffusive camera-based tracking with blur and localization errors

To streamline the presentation, we will use units where $\Delta t = 1$, use the subscript $t = 0, 1, 2, \ldots$ to denote discrete time dependence, and use the step length and localization variances, $\lambda_t = 2D_t\Delta t$ and $v_t = \varepsilon_t^2$, respectively. We allow both of them to be time-dependent, but assume them to be statistically independent and restrict the diffusion constant to be constant throughout each frame. Then, Eq. (8) reads

$$x_t = \int_0^1 f(t')y(t+t')\mathrm{d}t' + \sqrt{v_t}\xi_t, \tag{12}$$

where ξ_t are independent identically distributed (iid) N(0,1) variables, and $y(t)$ is the true trajectory of the particle being localized. The shutter distribution $f(t)$ is a probability density on $[0, 1]$, which describes the image acquisition process (for example $f(t) = 1$ for continuous acquisition), but neglects stochastic elements such as fluorophore blinking. It has the distribution function

$$F(t) = \int_0^t f(t')\mathrm{d}t'. \tag{13}$$

We divide $y(t)$ in two parts, the true positions y_t at the beginning of each frame, which evolve according to

$$y_{t+1} = y_t + \sqrt{\lambda_t}\eta_t, \tag{14}$$

where η_t are again iid N(0,1), and a conditional interpolating process between them, described by Brownian bridges [13]. Thus, for $0 \le t' \le 1$, we write

$$y(t+t') = y_t + t'(y_{t+1} - y_t) + \sqrt{\lambda_t}B_t(t'), \tag{15}$$

where B_t are a set of iid standard Brownian bridges. These are Gaussian processes on the interval [0,1], defined by

$$B_t(0) = B_t(1) = 0, \quad \langle B_t(t')\rangle = 0, \quad \langle B_t(t')B_t(t'')\rangle = t'(1-t''), \text{ for } t' \le t'', \tag{16}$$

and also independent on different intervals, so that $\left\langle B_i(t')B_j(t'')\right\rangle = 0$ if $i \neq j$. Substituting the interpolation formula Eq. (15) in the localization model Eq. (12), we get

$$x_t = y_t(1-\tau) + y_{t+1}\tau + \sqrt{v_t}\xi_t + \sqrt{\lambda_t}\int_0^1 f(t')B_t(t')\mathrm{d}t', \tag{17}$$

where we have introduced the shutter average, given by

$$\tau = \int_0^1 t f(t)\mathrm{d}t. \tag{18}$$

Using the properties of Brownian bridges, Eq. (16), one can show that the last integral in Eq. (17) is a Gaussian random variable with mean zero and variance

$$\beta \equiv \mathrm{Var}\left[\int_0^1 f(t')B_t(t')\mathrm{d}t'\right] = \tau(1-\tau) - R, \tag{19}$$

where R is the blur coefficient of Ref. [11], given by

$$R = \int_0^1 F(t)(1-F(t))\mathrm{d}t. \tag{20}$$

By assumption, v_t and B_t are statistically independent, and thus one can add up the noise in the measurement model and arrive at

$$x_t = y_t(1-\tau) + y_{t+1}\tau + \sqrt{v_t + \beta\lambda_t}\zeta_t, \tag{21}$$

where ζ_t are again iid N(0,1).

Constant exposure

An important class of shutter distribution are those that are constant during some fraction t_E of the each frame, and then zero, that is,

$$f(t) = \begin{cases} \frac{1}{t_\mathrm{E}}, & t \le t_\mathrm{E}, \\ 0, & t > t_\mathrm{E}, \end{cases} \tag{22}$$

which leads to

$$\tau = \frac{t_\mathrm{E}}{2}, \quad R = \frac{t_\mathrm{E}}{6}, \quad \beta = \frac{1}{4}t_\mathrm{E}(\frac{4}{3} - t_\mathrm{E}). \tag{23}$$

We see that $R \le \frac{1}{6}$ and $\tau \le \frac{1}{2}$, with maxima at continuous exposure ($t_\mathrm{E} = 1$). On the other hand, β has a maximum of $\frac{1}{9}$ at $t_\mathrm{E} = \frac{2}{3}$, and the value of $\frac{1}{12}$ at continuous exposure can only be further lowered when $t_\mathrm{E} < \frac{1}{3}$. It is unclear if this is significant, since with non-constant exposure there is some freedom in how the shutter distribution is defined. In this special case, one can for example always place it symmetrically in the interval and get $\tau = 0.5$ for all exposure times. We defer further investigations of this issue to future work.

Covariance relations

The covariance matrix for the steps $\Delta x_t = x_{t+1} - x_t$ can be found from Eqs. (14,21). With some manipulations, we get

$$\left\langle \Delta x_t^2 \right\rangle = (1-\tau)\lambda_t + \tau\lambda_{t+1} - (\lambda_{t+1} + \lambda_t)R + v_t + v_{t+1}, \tag{24}$$

$$\langle \Delta x_t \Delta x_{t+1} \rangle = \lambda_{t+1} R - v_{t+1}, \tag{25}$$

$$\langle \Delta x_t \Delta x_{t+t'} \rangle = 0, \text{ if } |t'| > 1, \tag{26}$$

where the expectations $\langle \cdot \rangle$ are understood to be over the noise distributions only. If we further assume simple diffusion, $\lambda_t = 2D\Delta t = const.$, and average over time as well, we recover covariance relations of the same form as Eq. (7),

$$\left\langle \Delta x_t^2 \right\rangle = 2D\Delta t(1-2R) + 2\langle v_t \rangle, \quad \langle \Delta x_t \Delta x_{t\pm1} \rangle = 2D\Delta t R - \langle v_t \rangle, \tag{27}$$

where the averages are now over time as well, and ε^2 is identified as the time-average $\langle v_t \rangle$. Thus, the covariance-based estimators of Ref. [1] should apply also to non-constant localization errors, if one uses the same time-averages for v_t and the step length moments.

Supplementary Note 10 Maximum likelihood estimator

For the case of a single trajectory of simple diffusion in one dimension, the likelihood for the diffusion constant follows from Eqs. 14 and 21,

$$p(x|\lambda) = \int \mathrm{d}y p(x|y,\lambda)p(y|\lambda), \tag{28}$$

$$p(y|\lambda) = \prod_{t=1}^{T} (2\pi\lambda)^{-\frac{1}{2}} \exp\Big[-\frac{(y_{t+1}-y_t)^2}{2\lambda}\Big], \tag{29}$$

$$p(x|y,\lambda) = \prod_{t=1}^{T} (2\pi(v_t+\beta\lambda))^{-\frac{1}{2}} \exp\Big[-\frac{1}{2}(v_t+\beta\lambda)^{-1}(x_t-(1-\tau)y_t-\tau y_{t+1})^2\Big], \tag{30}$$

where we neglected to supply a starting density for y_1, since the problem is translation invariant. The integral over the hidden path in Eq. (28) is a multivariate Gaussian and can be solved exactly in several ways [12]. Defining

$$\mathbf{y} = [y_1,\ y_2, \ldots, y_{T+1}]^\dagger, \quad \mathbf{x} = [x_1,\ x_2, \ldots, x_T]^\dagger, \tag{31}$$

where † denotes matrix transpose, we get

$$\begin{aligned} p(x|\lambda) = \int \mathrm{d}\mathbf{y} \exp\Big[-T\ln(2\pi) - \frac{T}{2}\ln(\lambda) - \frac{1}{2}\sum_{t=1}^{T}\ln(v_t+\beta\lambda) - \frac{1}{2}\big(\mathbf{y}^\dagger\Lambda\mathbf{y} - 2\mathbf{y}^\dagger W\mathbf{x} + \mathbf{x}^\dagger V\mathbf{x}\big)\Big] \\ = \exp\Big[-T\ln(2\pi) - \frac{T}{2}\ln(\lambda) - \frac{1}{2}\sum_{t=1}^{T}\ln(v_t+\beta\lambda)\Big] \\ \times \int \mathrm{d}\mathbf{y} \exp\Big[-\frac{1}{2}\big((\mathbf{y}-\Lambda^{-1}W\mathbf{x})^\dagger\Lambda(\mathbf{y}-\Lambda^{-1}W\mathbf{x}) + \mathbf{x}^\dagger(V - W^\dagger\Lambda^{-1}W)\mathbf{x}\big)\Big], \end{aligned} \tag{32}$$

where Λ, W, V are matrices whose elements are found by comparing terms. This is a multivariate Gaussian in $\mathbf{y}$, with mean value $\boldsymbol{\mu} = \Lambda^{-1}W\mathbf{x}$ and covariance matrix $\Sigma = \Lambda^{-1}$, and the marginalized likelihood is therefore given by

$$p(x|\lambda) = \exp\Big[-\frac{T-1}{2}\ln(2\pi) - \frac{T}{2}\ln(\lambda) - \frac{1}{2}\sum_{t=1}^{T}\ln(v_t+\beta\lambda) - \frac{1}{2}\mathbf{x}^\dagger(V - W^\dagger\Lambda^{-1}W)\mathbf{x} - \frac{1}{2}\ln|\Lambda|\Big]. \tag{33}$$

By comparing terms, we see that Λ is symmetric tridiagonal and positive definite, V is diagonal, and W has only non-zero elements on the diagonal and first upper diagonal, and so it is possible to compute the above matrix expression in linear time. To find the maximum likelihood estimate, we minimize $\ln p(x|\lambda)$ using standard optimization routines in Matlab. Two- or three-dimensional trajectories can be handled by treating them as independent observations. Missing points can be handled by setting the corresponding v_t-values to infinity.

Supplementary Note 11 Variational EM algorithm for diffusive HMM

Here, we extend the above diffusion model to include multiple diffusion constants. We start by writing down a diffusive HMM, which includes both the hidden path of the above diffusion model, but also a set of hidden states with different diffusion constants, that evolve as a discrete Markov process. Similar models (that however did not include explicit motion blur effects), have previously been solved by stochastic EM algorithms [14, 15]. Here, we instead describe a deterministic variational approach.

In the rest of this section, we proceed as follows: We start by specify the diffusive HMM model for a single 1-dimensional trajectory. We then outline the variational EM approach, derive high level update equations, and describe the procedure for re-estimating localized positions. However, we do not give a detailed derivation of all steps in the algorithm, as large parts of it closely resembles previously published derivations of variational algorithms for HMMs [5, 6].

Model

In addition to the measured ($\mathbf{x}$) and true ($\mathbf{y}$) positions, we include a hidden state trajectory $\mathbf{s} = [s_1, s_2, \ldots, s_T]$, such that s_t determines the diffusion constant on the interval $[t, t+1]$. The hidden states are numbered from 1 to N, and evolve according to a Markov process with transition matrix A and initial state probability $\boldsymbol{\pi}$. The vector of state-dependent step-length variances is denoted $\boldsymbol{\lambda} = [\lambda_1, \lambda_2, \ldots, \lambda_N]$. For a single 1-dimensional trajectory, this leads to a complete data likelihood of the form

$$p(\mathbf{x}, \mathbf{y}, \mathbf{s}|\boldsymbol{\lambda}, A, \boldsymbol{\pi}) = p(\mathbf{x}|\mathbf{y}, \mathbf{s}, \boldsymbol{\lambda})p(\mathbf{y}|\mathbf{s}, \boldsymbol{\lambda})p(\mathbf{s}|A, \boldsymbol{\pi}), \tag{34}$$

with factors

$$p(\mathbf{s}|A, \boldsymbol{\pi}) = \prod_{m=1}^{N} \pi_m^{\delta_{m,s_1}} \prod_{t=2}^{T} \prod_{i,j=1}^{N} A_{ij}^{\delta_{is_t}\delta_{js_{t+1}}}, \tag{35}$$

$$p(\mathbf{y}|\mathbf{s}, \boldsymbol{\lambda}) = \prod_{t=1}^{T} \prod_{j=1}^{N} (2\pi\lambda_j)^{-\frac{1}{2}\delta_{js_t}} \exp\left[-\delta_{js_t}\frac{(y_{t+1} - y_t)^2}{2\lambda_j}\right], \tag{36}$$

$$p(\mathbf{x}|\mathbf{y}, \mathbf{s}, \boldsymbol{\lambda}) = \prod_{t=1}^{T} \prod_{j=1}^{N} (2\pi(v_t + \beta\lambda_j))^{-\frac{1}{2}\delta_{js_t}} \exp\left[-\delta_{js_t}\frac{(x_t - (t-\tau)y_t - \tau y_{t+1})^2}{2(v_t + \beta\lambda_j)}\right]. \tag{37}$$

Variational EM approach

We would like to perform maximum-likelihood inference of the model parameters, which means maximizing the likelihood with latent variables $\mathbf{s}, \mathbf{y}$ integrated out,

$$L(A, \boldsymbol{\pi}, \boldsymbol{\lambda}) = \int d\mathbf{y} \sum_{\mathbf{s}} p(\mathbf{x}|\mathbf{y}, \mathbf{s}, \boldsymbol{\lambda})p(\mathbf{y}|\mathbf{s}, \boldsymbol{\lambda})p(\mathbf{s}|A, \boldsymbol{\pi}). \tag{38}$$

Since this problem is intractable, we make a variational approximation [16], meaning we approximate $\ln L$ with a lower bound

$$\ln L = \ln \int d\mathbf{y} \sum_{\mathbf{s}} p(\mathbf{x}|\mathbf{y}, \mathbf{s}, \boldsymbol{\lambda})p(\mathbf{y}|\mathbf{s}, \boldsymbol{\lambda})p(\mathbf{s}|A, \boldsymbol{\pi}) \geq \int d\mathbf{y} \sum_{\mathbf{s}} q(\mathbf{s})q(\mathbf{y}) \ln \frac{p(\mathbf{x}|\mathbf{y}, \mathbf{s}, \boldsymbol{\lambda})p(\mathbf{y}|\mathbf{s}, \boldsymbol{\lambda})p(\mathbf{s}|A, \boldsymbol{\pi})}{q(\mathbf{s})q(\mathbf{y})} \equiv F, \tag{39}$$

where the inequality follows from Jensen's inequality. Here, $q(\mathbf{s})$, $q(\mathbf{y})$ are arbitrary variational distributions that need to be optimized together with the model parameters to achieve the tightest lower bound, and can be used for approximate inference about the latent variables. In particular, it turns out that the optimal variational distributions approximate the posterior distribution of $\mathbf{y}, \mathbf{s}$ in the sense of minimizing a Kullback-Leibler divergence [16]. We use gradient descent for the optimization, that is, we iteratively optimize each variational distribution and the parameters with the others fixed, which leads to an EM-type algorithm.

To optimize F w.r.t. the variational distributions, we set the functional derivatives of F to zero and use Lagrange multipliers to enforce normalization. After some work, one arrives at

$$\ln q(\mathbf{s}) = -\ln Z_s + \langle \ln p(\mathbf{x}|\mathbf{y}, \mathbf{s}, \boldsymbol{\lambda})\rangle_{q(\mathbf{y})} + \langle \ln p(\mathbf{y}|\mathbf{s}, \boldsymbol{\lambda})\rangle_{q(\mathbf{y})} + \ln p(\mathbf{s}|A, \boldsymbol{\pi}), \tag{40}$$

$$\ln q(\mathbf{y}) = -\ln Z_y + \langle \ln p(\mathbf{x}|\mathbf{y}, \mathbf{s}, \boldsymbol{\lambda})\rangle_{q(\mathbf{s})} + \langle \ln p(\mathbf{y}|\mathbf{s}, \boldsymbol{\lambda})\rangle_{q(\mathbf{s})} + \underbrace{\langle \ln p(\mathbf{s}|A, \boldsymbol{\pi})\rangle_{q(\mathbf{s})}}_{\text{independent of } \mathbf{y}}, \tag{41}$$

where $Z_{s,y}$ are normalization constants originating from the Lagrange multipliers, and $\langle\cdot\rangle_f$ denotes an expectation value computed with respect to the distribution f. As it turns out, these equations are individually tractable, and results in $q(\mathbf{y})$ being a multivariate Gaussian, and $q(\mathbf{s})$ adopting the standard HMM form amenable to efficient forward-backward iterations. For the initial state and transition probability parameters, the only dependence in F comes from $p(\mathbf{s}|A,\boldsymbol{\lambda})$, and leads to the classical Baum-Welch reestimation formulae [17]. However, optimizing F w.r.t. step length variances does not lead to a tractable update equation, and we are instead forced to optimize the λ_j-dependent parts of F numerically, that is,

$$\lambda_j = \operatorname{argmax}_{\lambda_j}\Big[\langle\ln p(\mathbf{x}|\mathbf{y},\mathbf{s},\boldsymbol{\lambda})\rangle_{q(\mathbf{y})q(\mathbf{s})} + \langle\ln p(\mathbf{y}|\mathbf{s},\boldsymbol{\lambda})\rangle_{q(\mathbf{y})q(\mathbf{s})}\Big]. \tag{42}$$

The lower bound

The lower bound can be computed as well, and gets a particularly simple form just after the update of $q(\mathbf{s})$. Substituting the update equation Eq. (40) into the expression for F, Eq. (39), we get

$$F = \int d\mathbf{y}\sum_{\mathbf{s}} q(\mathbf{s})q(\mathbf{y})\ln\Big[\ln p(\mathbf{x}|\mathbf{y},\mathbf{s},\boldsymbol{\lambda}) + \ln p(\mathbf{y}|\mathbf{s},\boldsymbol{\lambda}) + \ln p(\mathbf{s},A,\boldsymbol{\pi})$$
$$+ \ln Z_s - \langle\ln p(\mathbf{x}|\mathbf{y},\mathbf{s},\boldsymbol{\lambda}) + \ln p(\mathbf{y}|\mathbf{s},\boldsymbol{\lambda}) + \ln p(\mathbf{s},A,\boldsymbol{\pi})\rangle_{q(\mathbf{y})} - \ln q(\mathbf{y})\Big] = \ln Z_s - \langle\ln q(\mathbf{y})\rangle_{q(\mathbf{y})}. \tag{43}$$

Here, $\ln Z_s$ is the normalization constant of $q(\mathbf{s})$ that can be computed as part of the forward-backward iteration, and since (as we noted above) $q(\mathbf{y})$ is a multivariate Gaussian with dimension $T+1$, we get

$$-\langle\ln q(\mathbf{y})\rangle_{q(\mathbf{y})} = \frac{d}{2}(T+1)(1+\ln(2\pi)) + \frac{1}{2}\ln|\Sigma|, \tag{44}$$

where Σ is the covariance matrix of $q(\mathbf{y})$. The inverse of Σ is analogous to the matrix Λ appearing in the single-state diffusion estimator, Eqs. (32,33), and in particular Σ^{-1} is also symmetric, tridiagonal, and positive definite, and thus the determinant $\ln|\Sigma| = -\ln|\Sigma^{-1}|$ can be robustly computed in linear time. Since the EM algorithm approximates the parameter likelihood, the lower bound cannot be used for model selection as in the case of variational maximum evidence calculations [5, 6, 18]. However, it is still useful for numerical convergence control, and possibly for model selection together with some complexity penalty such as the Bayesian or Akaike information criterion [19].

Refined positions

An additional use for the HMM analysis is to use it to refine the localizations. Since the HMM pools information about many spots, it is in principle possible to beat the Cramér-Rao lower bound for single image localizations in this way. To set up the refinement problem, we refer back to Eq. (17) and the different contributions to the observed position $x_t = z_t + \sqrt{v_t}\xi_t$, where

$$z_t = y_t(1-\tau) + y_{t+1}\tau + \sqrt{\lambda_{s_t}}\int_0^1 f(t')B_t(t')dt', \tag{45}$$

is the motion-averaged position that the localization algorithm tries to estimate (according to this model). To refine the localization, we compute the posterior density of z_t, that is, $p(z_t|\mathbf{x},\theta)$, where we use θ to denote all the model parameters.

We start by recalling that the Brownian bridge integral in Eq. (45) is Gaussian with mean zero and variance $\beta\lambda_{s_t}$, and using the compact notation $\overline{y_t} = (1-\tau)y_t + \tau y_{t+1}$, we therefore have

$$p(z_t|\mathbf{y},\mathbf{s},\theta) = p(z_t|\overline{y}_t,s_t,\theta) = N(\overline{y}_t,\beta\lambda_{s_t}), \tag{46}$$

where $N(a,b)$ denotes a Gaussian density with mean a and variance b. Furthermore, the localization uncertainty is also assumed Gaussian and independent of the underlying kinetics:

$$p(x_t|\mathbf{z},\mathbf{y},\mathbf{s},\theta) = p(x_t|z_t) = N(z_t,v_t). \tag{47}$$

Applying Bayes theorem to these relations, we get

$$p(z_t|x_t,\mathbf{y},\mathbf{s},\theta) = \frac{p(x_t|z_t)p(z_t|\mathbf{y},\mathbf{s},\theta)}{p(x_t|\mathbf{y},\mathbf{s},\theta))} = \frac{N(z_t,v_t)N(\overline{y}_t,\beta\lambda_{s_t})}{N(\overline{y}_t,\beta\lambda_{s_t}+v_t)} = N\Big(\frac{v_t\overline{y}_t+\beta\lambda_{s_t}x_t}{v_t+\beta\lambda_{s_t}},\frac{\beta\lambda_{s_t}v_t}{v_t+\beta\lambda_{s_t}}\Big). \tag{48}$$

The predictive distribution is finally given by marginalizing $p(z_t|x_t, \mathbf{y}, \mathbf{s}, \theta)$ over the posterior for $\mathbf{y}, \mathbf{s}$. Using the variational distribution, this means

$$p(z_t|\mathbf{x}, \theta) \approx \left\langle N\Big(\frac{v_t\bar{y}_t + \beta\lambda_{s_t}x_t}{v_t + \beta\lambda_{s_t}}, \frac{\beta\lambda_{s_t}v_t}{v_t + \beta\lambda_{s_t}}\Big)\right\rangle_{q(\mathbf{y})q(\mathbf{s})}. \tag{49}$$

In particular, the posterior mean of z_t is then given by

$$\langle z_t|\mathbf{x}, \theta\rangle \approx \left\langle \frac{v_t\bar{y}_t + \beta\lambda_{s_t}x_t}{v_t + \beta\lambda_{s_t}}\right\rangle_{q(\mathbf{y})q(\mathbf{s})} = \left\langle \frac{(1-\tau)\mu_t + \tau\mu_{t+1} + \frac{\beta\lambda_{s_t}}{v_t}x_t}{1 + \frac{\beta\lambda_{s_t}}{v_t}}\right\rangle_{q(\mathbf{s})}, \tag{50}$$

which we will use as our estimator for refining the localizations. Here, $\mu_t = \langle y_t\rangle_{q(\mathbf{y})}$ is the variational mean value. The variational average over hidden states is done numerically.

Supplementary References

[1] Christian L. Vestergaard, Paul C. Blainey, and Henrik Flyvbjerg. Optimal estimation of diffusion coefficients from single-particle trajectories. *Phys. Rev. E*, 89(2):022726, February 2014. doi: 10.1103/PhysRevE.89.022726.

[2] Kim I. Mortensen, L. Stirling Churchman, James A. Spudich, and Henrik Flyvbjerg. Optimized localization analysis for single-molecule tracking and super-resolution microscopy. *Nat. Meth.*, 7(5):377–381, 2010. doi: 10.1038/nmeth.1447.

[3] Sean R Eddy. What is Bayesian statistics? *Nat. Biotech.*, 22(9):1177–1178, 2004. doi: 10.1038/nbt0904-1177.

[4] Jan-Willem van de Meent, Jonathan E. Bronson, Chris H. Wiggins, and Ruben L. Gonzalez. Empirical Bayes methods enable advanced population-level analyses of single-molecule FRET experiments. *Biophys J*, 106(6):1327–1337, 2014. doi: 10.1016/j.bpj.2013.12.055.

[5] Stephanie Johnson, Jan-Willem van de Meent, Rob Phillips, Chris H. Wiggins, and Martin Lindén. Multiple LacI-mediated loops revealed by Bayesian statistics and tethered particle motion. *Nucl. Acids Res.*, 42(16):10265–10277, 2014. doi: 10.1093/nar/gku563.

[6] Fredrik Persson, Martin Lindén, Cecilia Unoson, and Johan Elf. Extracting intracellular diffusive states and transition rates from single-molecule tracking data. *Nat. Meth.*, 10(3):265–269, 2013. doi: 10.1038/nmeth.2367.

[7] Sheng Liu, Emil B. Kromann, Wesley D. Krueger, Joerg Bewersdorf, and Keith A. Lidke. Three dimensional single molecule localization using a phase retrieved pupil function. *Opt. Express*, 21(24):29462–29487, 2013. doi: 10.1364/OE.21.029462.

[8] Hendrik Deschout, Kristiaan Neyts, and Kevin Braeckmans. The influence of movement on the localization precision of sub-resolution particles in fluorescence microscopy. *J. Biophotonics*, 5(1):97–109, 2012. doi: 10.1002/jbio.201100078.

[9] Gareth Loy and Alexander Zelinsky. Fast radial symmetry for detecting points of interest. *IEEE Trans. Pattern Anal. Mach. Intell.*, 25(8):959–973, 2003. doi: 10.1109/TPAMI.2003.1217601.

[10] David J. C. MacKay. Choice of basis for Laplace approximation. *Mach. Learn.*, 33(1):77–86, 1998. doi: 10.1023/A:1007558615313.

[11] Andrew J. Berglund. Statistics of camera-based single-particle tracking. *Phys. Rev. E*, 82(1):011917, 2010. doi: 10.1103/PhysRevE.82.011917.

[12] Peter K. Relich, Mark J. Olah, Patrick J. Cutler, and Keith A. Lidke. Estimation of the diffusion constant from intermittent trajectories with variable position uncertainties. *Phys. Rev. E*, 93(4):042401, 2016. doi: 10.1103/PhysRevE.93.042401.

[13] Winston C. Chow. Brownian bridge. *WIREs Comp. Stat.*, 1(3):325–332, 2009. doi: 10.1002/wics.38.

[14] Trevor T. Ashley and Sean B. Andersson. Method for simultaneous localization and parameter estimation in particle tracking experiments. *Phys. Rev. E*, 92(5):052707, 2015. doi: 10.1103/PhysRevE.92.052707.

[15] Jason Bernstein and John Fricks. Analysis of single particle diffusion with transient binding using particle filtering. *J. Theor. Biol.*, 401:109–121, 2016. doi: 10.1016/j.jtbi.2016.04.013.

[16] Christopher Bishop. *Pattern recognition and machine learning*. Springer, New York, 2006.

[17] L. R. Rabiner and B. H. Juang. An introduction to hidden Markov models. *IEEE ASSp Magazine*, pages 4–16, January 1986.

[18] Jonathan E. Bronson, Jingyi Fei, Jake M. Hofman, Ruben L. Gonzalez Jr., and Chris H. Wiggins. Learning rates and states from biophysical time series: A Bayesian approach to model selection and single-molecule FRET data. *Biophys. J.*, 97(12):3196–3205, 2009. doi: 10.1016/j.bpj.2009.09.031.

[19] Kenneth P. Burnham and David R. Anderson. *Model Selection and Multimodel Inference: A Practical Information-Theoretic Approach*. Springer, 2013.
