# Supplementary figures and images for: Pointwise error estimates in localization microscopy
Source: Nat Commun. 2017 May 3;8:15115. doi: 10.1038/ncomms15115 (PMC5418599; doi:10.1038/ncomms15115)

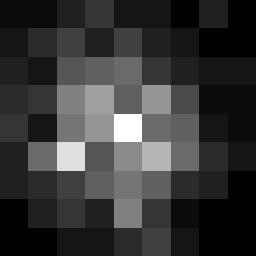

Supplement: Supplementary Data 1 — Collection of Matlab scripts to illustrate how we generated and analyzed synthetic data. See readme.txt for dependency information. [file ncomms15115-s2.zip › SPT_examples/ex3_dot_detection/FRST/Examples/input_dot.png]

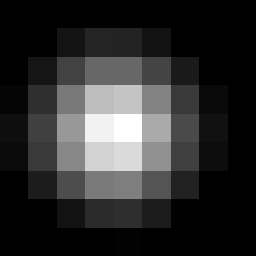

Supplement: Supplementary Data 1 — Collection of Matlab scripts to illustrate how we generated and analyzed synthetic data. See readme.txt for dependency information. [file ncomms15115-s2.zip › SPT_examples/ex3_dot_detection/FRST/Examples/results_PP.png]

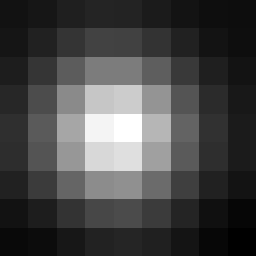

Supplement: Supplementary Data 1 — Collection of Matlab scripts to illustrate how we generated and analyzed synthetic data. See readme.txt for dependency information. [file ncomms15115-s2.zip › SPT_examples/ex3_dot_detection/FRST/Examples/results_S.png]

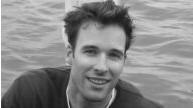

Supplement: Supplementary Data 1 — Collection of Matlab scripts to illustrate how we generated and analyzed synthetic data. See readme.txt for dependency information. [file ncomms15115-s2.zip › SPT_examples/ex3_dot_detection/FRST/g.jpg]

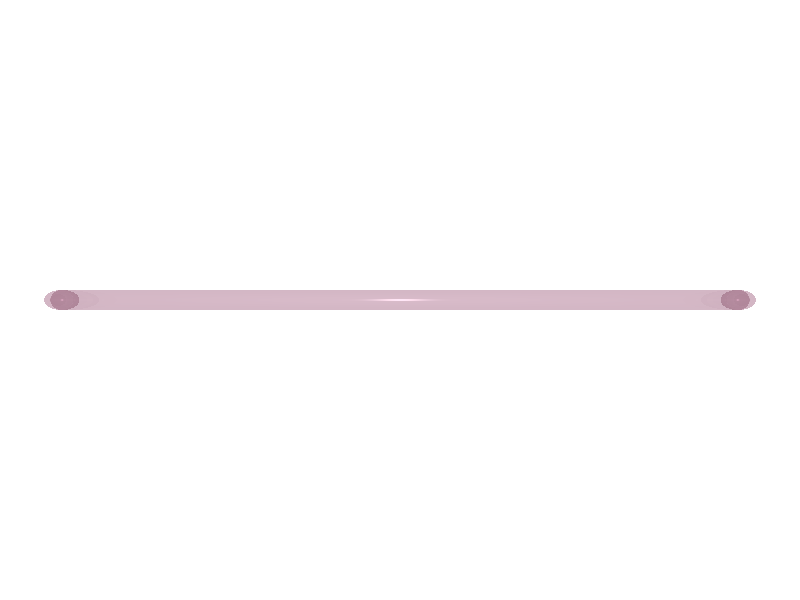

Supplement: Supplementary Data 1 — Collection of Matlab scripts to illustrate how we generated and analyzed synthetic data. See readme.txt for dependency information. [file ncomms15115-s2.zip › SPT_examples/mesoRDmodel_D100_longCells/geometry.png]

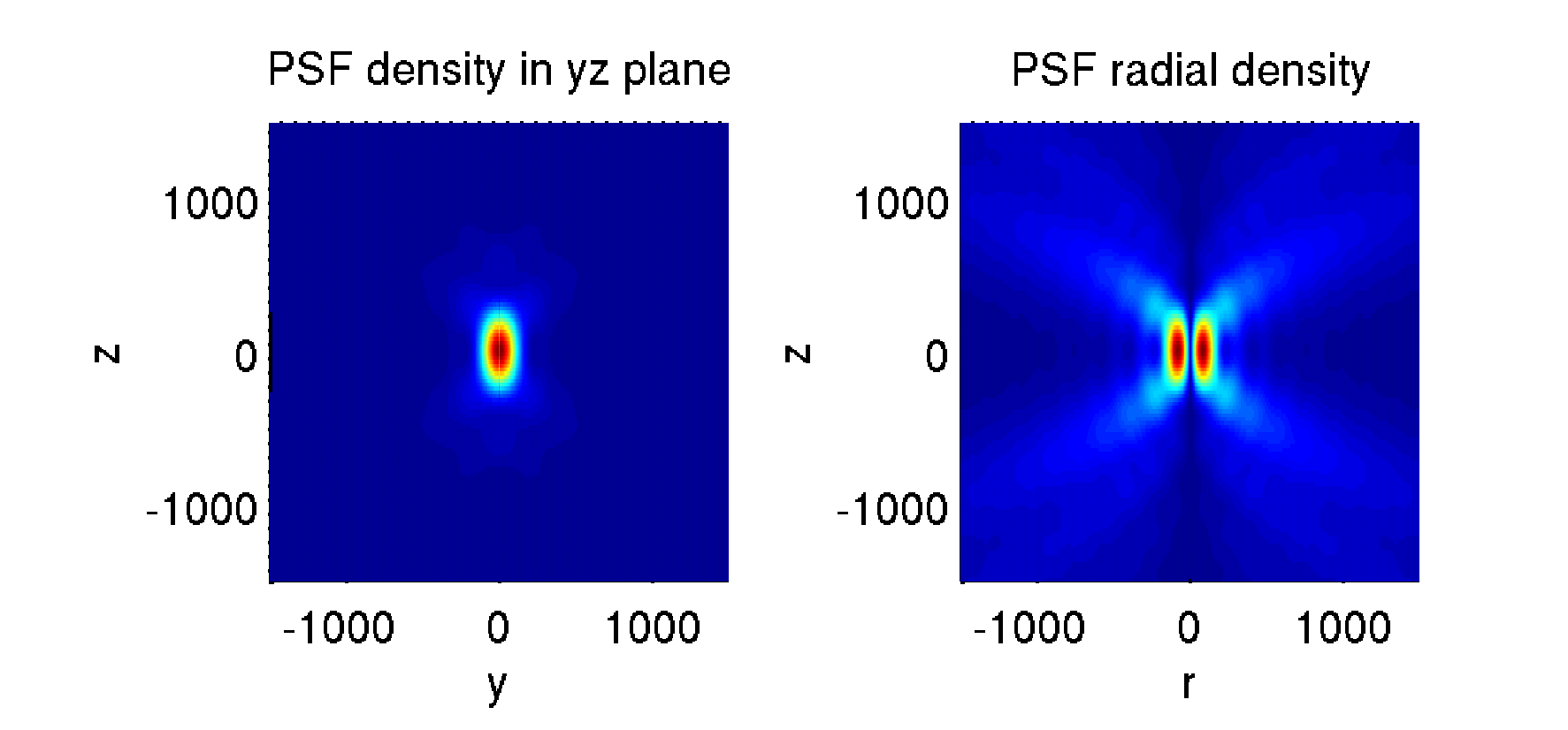

Supplement: Supplementary Data 1 — Collection of Matlab scripts to illustrate how we generated and analyzed synthetic data. See readme.txt for dependency information. [file ncomms15115-s2.zip › SPT_examples/psfModels/psf_RW_514nm_NA149_ni1515.png]

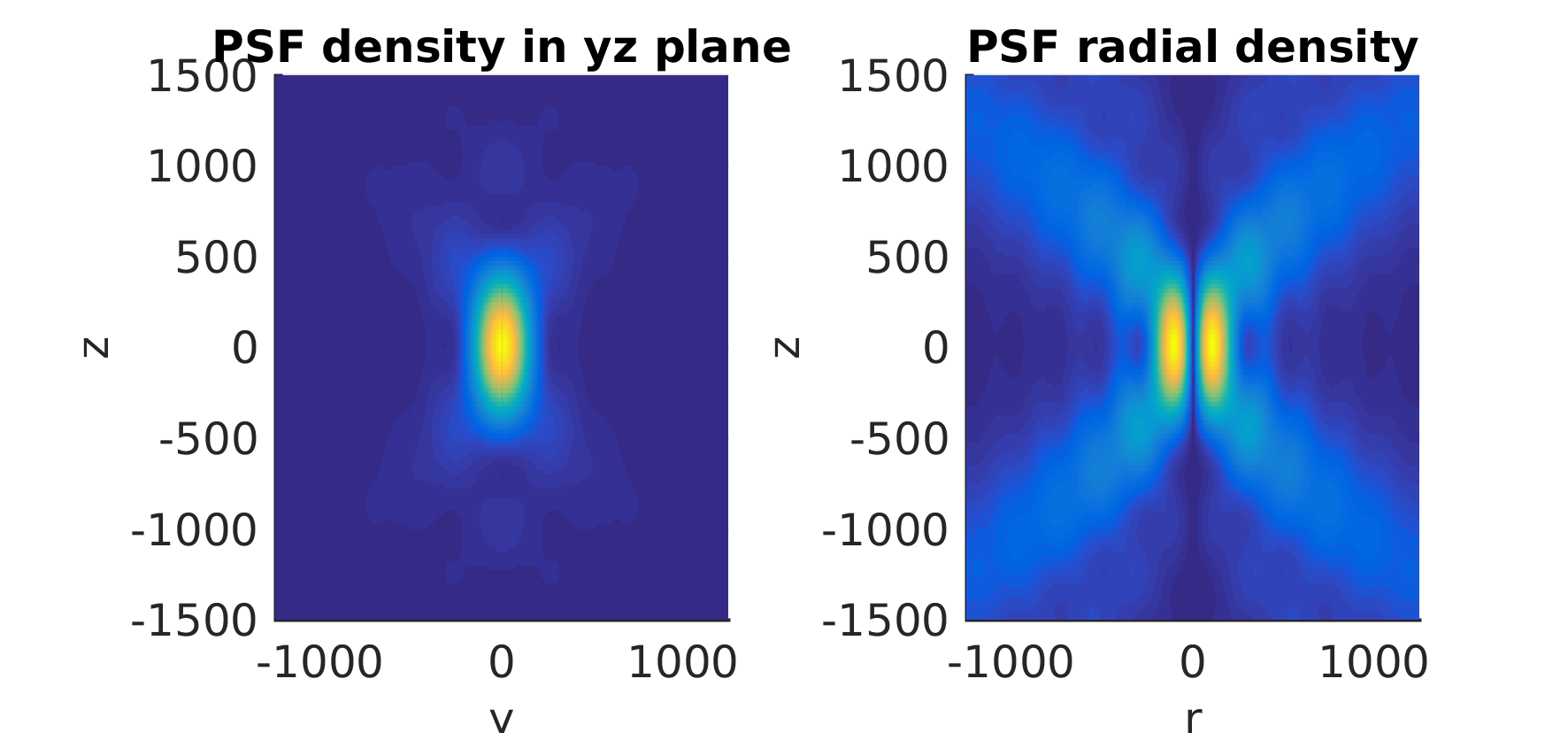

Supplement: Supplementary Data 1 — Collection of Matlab scripts to illustrate how we generated and analyzed synthetic data. See readme.txt for dependency information. [file ncomms15115-s2.zip › SPT_examples/psfModels/psf_RW_639nm_NA14_ni1515.png]

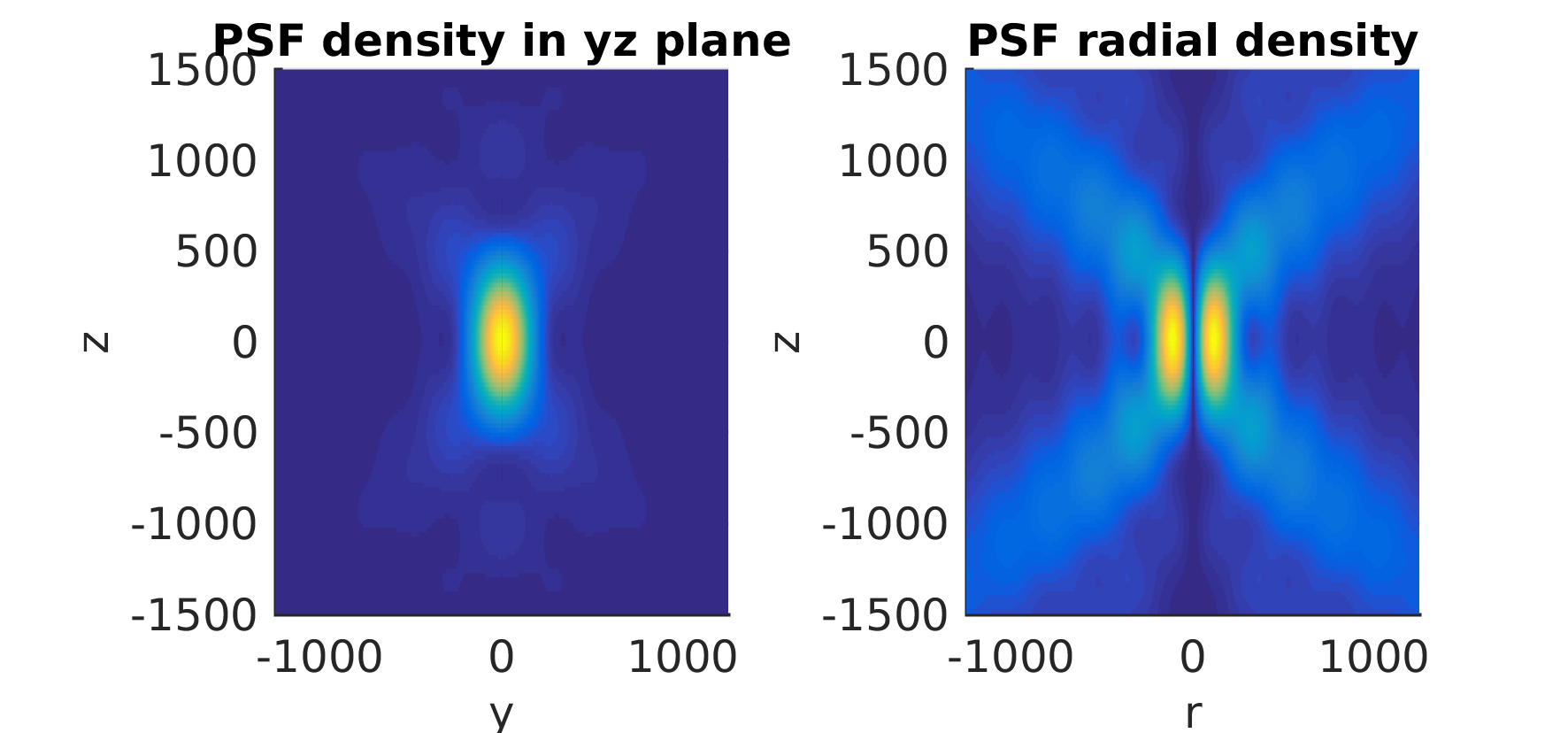

Supplement: Supplementary Data 1 — Collection of Matlab scripts to illustrate how we generated and analyzed synthetic data. See readme.txt for dependency information. [file ncomms15115-s2.zip › SPT_examples/psfModels/psf_RW_680nm_NA14_ni1515.png]
